# Integrative network analysis of transcriptomics data reveals potential prognostic biomarkers for colorectal cancer

**DOI:** 10.1002/cam4.7391

**Published:** 2024-06-14

**Authors:** Mohita Mahajan, Angshuman Sarkar, Sukanta Mondal

**Affiliations:** ^1^ Department of Biological Sciences Birla Institute of Technology and Science Pilani, K.K. Birla Goa campus Goa India

**Keywords:** gene co‐expression network, gene regulatory network, KEGG pathways, pathway cross‐talk, prognostic biomarkers, reactome pathways

## Abstract

**Introduction:**

Cross‐talk among biological pathways is essential for normal biological function and plays a significant role in cancer progression. Through integrated network analysis, this study explores the significance of pathway cross‐talk in colorectal cancer (CRC) development at both the pathway and gene levels.

**Methods:**

In this study, we integrated the gene expression data with domain knowledge to construct state‐dependent pathway cross‐talk networks. The significance of the genes involved in pathway cross‐talk was assessed by analyzing their association with cancer hallmarks, disease‐gene relation, genetic alterations, and survival analysis. We also analyzed the gene regulatory network to identify the dysregulated genes and their role in CRC progression.

**Results:**

Cross‐talk was observed between immune‐related pathways and pathways associated with cell communication and signaling. The PTPRC gene was identified as a mediator, facilitating interactions within the immune system and other signaling pathways. The rewired interactions of ITGA7 were identified as influential in the epithelial‐mesenchymal transition in CRC. This study also highlighted the crucial link between cell communication and vascular smooth muscle contraction pathway in CRC progression. The survival analysis of identified gene clusters showed their significant prognostic value in distinguishing high‐risk from low‐risk CRC groups, and L1000CDS2 revealed seven potential drug molecules in CRC. Nine dysregulated genes (CTNNB1, EP300, JUN, MYC, NFKB1, RELA, SP1, STAT1, and TP53) emerge as transcription factors acting as common regulators across various pathways.

**Conclusions:**

This study highlights the crucial role of pathway cross‐talk in CRC progression and identified the potential prognostic biomarkers and potential drug molecules.

## Graphical abstract

Rewired KEGG pathway cross‐talk network from the normal to the CRC state and their association with cancer hallmarks.

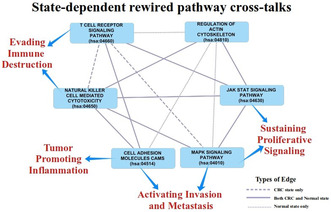

## 1 INTRODUCTION

A biological pathway is a sequence of interactions among expressed genes that regulates various biological processes, including cell growth, differentiation, migration, and cell death. In different biological contexts, these pathways coordinate or interact with one another to perform the specific biological function, a process known as pathway cross‐talk. Pathway cross‐talk occurs through a variety of mechanisms, including transcriptional regulation, the presence of shared components, and protein–protein interactions.^1^, ^2^ The phenomena of pathway cross‐talk is crucial in understanding the development of diseases, particularly in the context of cancer. Cancer, in essence, is the result of multiple alterations in the signaling pathways. Studies have observed a link of pathway cross‐talk in tumor growth and the development of resistance to therapeutic strategies. For instance, interactions between the Mitogen‐Activated Protein Kinase (MAPK) pathway and other cancer‐related pathways, such as Transforming Growth Factor‐β (TGF‐β) and NOTCH pathways are examples of such cross‐talk.^3^ Thus, to enhance diagnostic accuracy, prognostic capability, and overall clinical outcomes, we need to have a better understanding of the disease mechanism at both the systems and molecular levels.

Several studies have been conducted to identify and understand the cross‐talk among pathways. Initially, the pathways with the shared component were considered to be cross‐talked. However, as the boundaries of biological pathways are knowledge‐driven, they may interact with other pathways, even without shared components. Therefore, to address this complexity, PPIs (protein–protein interactions), gene expression, and gene ontology information were integrated with the pathways information.^2^ Sun et al.^4^ analyzed the pathway cross‐talk by integrating the differentially expressed genes (DEGs) information with the gene set enrichment analysis (GSEA) to identify the crucial pathways in breast cancer. Moon et al.^5^ introduce PINTnet, a method for constructing pathway interaction networks by integrating the PPIs, DEGs, and pathway information. The interactions between pathways were identified by considering both topological features and gene expression patterns. Joshi et al.^6^ proposed a route‐based approach to identify the pathway cross‐talk in diverse cancer cohorts, including the colon adenocarcinoma. This study focused on the transcription factors (TFs) in which cross‐talk routes start from the TFs of one pathway to the ligand of another pathway. Liu et al.^7^ presented a cross‐talk‐based pathway enrichment analysis by integrating the pathways information with the TF‐gene regulation and PPIs to identify cancer risk pathways. Among cancer types, colorectal cancer (CRC) is one of the leading causes of cancer‐related mortality worldwide (https://gco.iarc.fr/). The dysregulation of several signaling pathways, including Wnt, NF‐κB, and the NOTCH pathway, notably activated by the APC (Adenomatous polyposis coli) mutant, has been well‐documented in the development of CRC.^8^ Wang et al.^9^ integrated the PPIs network with the pathway information to identify the dysregulated pathways in CRC. Gene co‐expression analysis serves as a valuable approach for detecting clusters of genes that are co‐regulated or may be involved in related biological activities. By analyzing the alterations in gene co‐expression patterns under different conditions, studies have identified the pathways that are dysregulated under certain conditions.^10^, ^11^

In this study, the integration of state‐dependent gene co‐expression networks with pathway information was conducted to explore and understand the alterations in pathway cross‐talk from a normal state to a CRC state. To get molecular insights into the pathway cross‐talks, we further analyzed these pathway cross‐talks at the gene level and evaluated the biological significance of these genes by analyzing their association with cancer hallmarks, mutation, and disease. Survival analysis was performed to understand the impact of the selected genes in CRC progression. The L1000CDS^2^ database (L1000 Characteristic Direction Signature Search Engine) was explored to identify potential therapeutic drug candidates associated with genes showing significant prognostic value. The directed gene regulatory networks integrated with the gene expression data were also analyzed to identify the most dysregulated genes in the CRC state. The identified key pathways and genes in the present study may expand our understanding of the molecular mechanisms underlying CRC progression and may provide improved therapeutic strategies for CRC management.

## 2 METHODS AND MATERIALS

**FIGURE 1:**
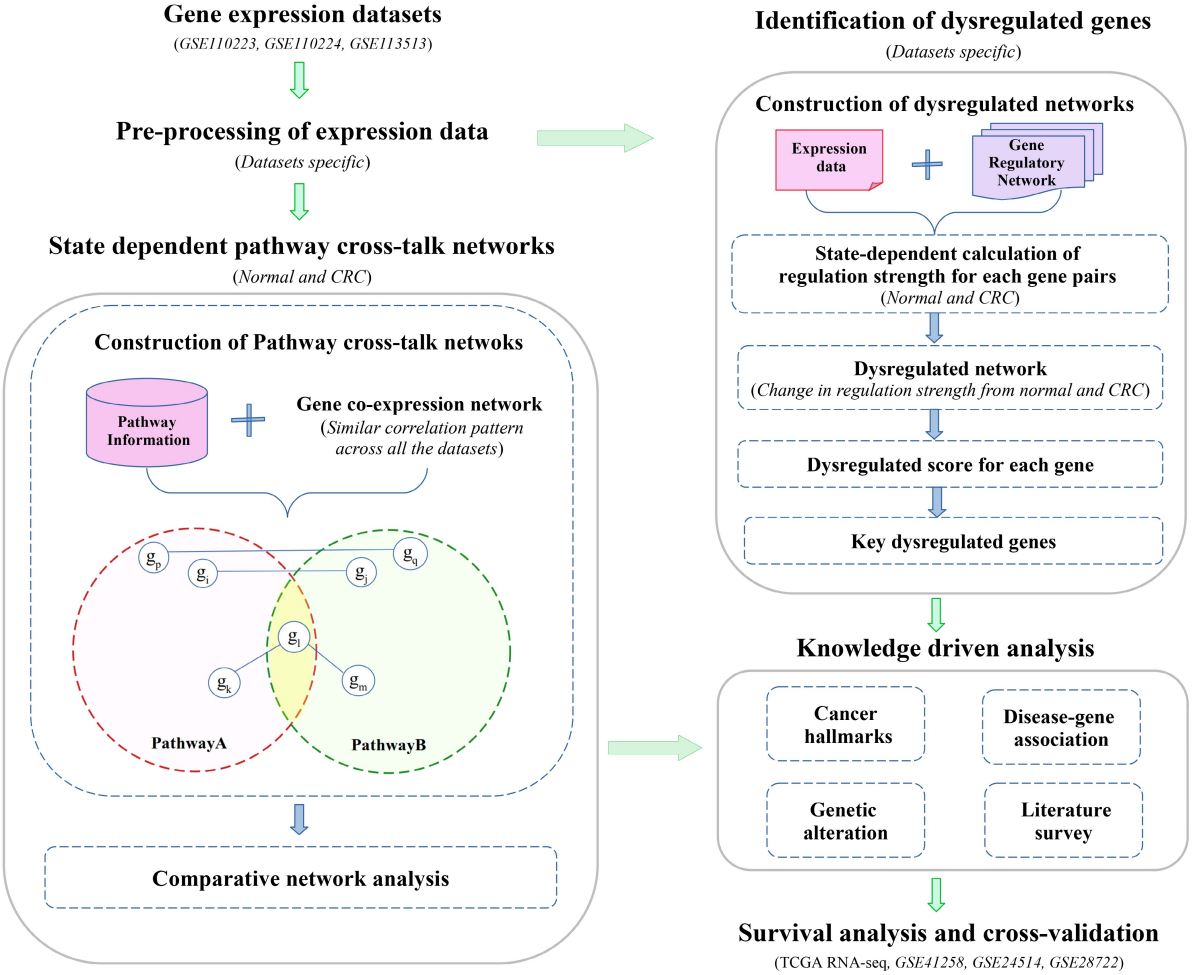
Overview of the workflow used in this study.

The workflow of integrated bioinformatics analysis used in this study is presented in Figure 1.

### 2.1 Selection of gene expression datasets and human biological pathways

The expression datasets containing the CRC and their adjacent normal tissue samples were searched in the Gene Expression Omnibus database (GEO, https://www.ncbi.nlm.nih.gov/geo/). Three datasets, namely, GSE110223, GSE110224, and GSE113513, were selected based on the following criteria: (i) tissue samples must be human CRC and normal samples, (ii) total sample size in each dataset should be >20, (iii) tissue with no drug treatment or knocking out a gene. The detailed information about the samples can be found in Table (Table 1). All the selected datasets were preprocessed independently using the robust multi‐array average (RMA) algorithm.^17^ We considered the common genes across all three selected datasets for further study. Limma (linear models for microarray data) package in the R programming software^18^ was used to analyze the fold change (log2FC) in gene expression between normal and CRC states with a statistical significance with a *p*‐value < 0.01.^19^

**TABLE 1:** The summary of the selected transcriptomic datasets used in this study.

Dataset	Objective	Number of normal samples	Number of CRC samples	References
GSE110223	Key genes and gene clusters (prognostic biomarker) identification	13	13	^12^
GSE110224	Key genes and gene clusters (prognostic biomarker) identification	17	17	^12^
GSE113513	Key genes and gene clusters (prognostic biomarker) identification	14	14	^13^
GSE24514	Key genes validation	15	34	^14^
GSE41258	Prognostic performance and Key genes validation	54	244	^15^
TCGA‐COADREAD	Prognostic performance and key genes validation	50	467	TCGA
GSE28722	Prognostic performance	‐	125	^16^

In this study, we selected the pathway information from the KEGG (Kyoto Encyclopedia of Genes and Genomes, https://www.genome.jp/kegg/pathway.html) and Reactome (https://reactome.org/) databases. Pathways, including the gene sets, were retrieved from the C2: Canonical Pathway collection of the MSigDB (Molecular Signature Database, https://www.gsea‐msigdb.org/gsea/msigdb). The pathways were further selected based on the following criteria: (i) pathways with a minimum of five genes appeared in the selected expression datasets, (ii) exclude the pathways belonging to human disease and drug development to reduce the pathway redundancy, and (iii) at least two overlapping genes among two pathways. The pathway analysis was performed independently for both the KEGG and Reactome pathways.

Besides the above three datasets, GSE24514, GSE28722, GSE41258, and TCGA RNA‐Seq were used to further analyze and cross‐validate the prognostic significance of the selected genes. The TCGA RNA‐Seq dataset comprised 50 paired normal and CRC samples obtained from the Genomic Data Commons Data Portal (https://portal.gdc.cancer.gov/). Normalization and analysis of log2FC for RNA‐seq data were conducted using the DESeq2 method.

### 2.2 Construction of state‐dependent pathway cross‐talk networks

As pathways as well, genes interact with each other under certain conditions; therefore, we constructed the pathway cross‐talk networks for normal and CRC states separately. For this, we integrated the state‐dependent gene co‐expression with the selected pathways. The gene co‐expression networks were independently constructed for normal and CRC states in all the selected datasets. The Pearson correlation coefficient (PCC) was calculated for gene pairs within each state using the HMISC package in R (https://hbiostat.org/R/Hmisc/). This study considers the gene pairs with PCC ≥ |0.7| and *p*‐value < 0.05 as co‐expressed genes. The final co‐expression network included gene pairs that exhibit a similar correlation pattern, either positive or negative, across all three datasets. Then, co‐expressed gene pairs were mapped with the selected pathways to construct the state‐dependent pathway cross‐talks.

**FIGURE 2:**
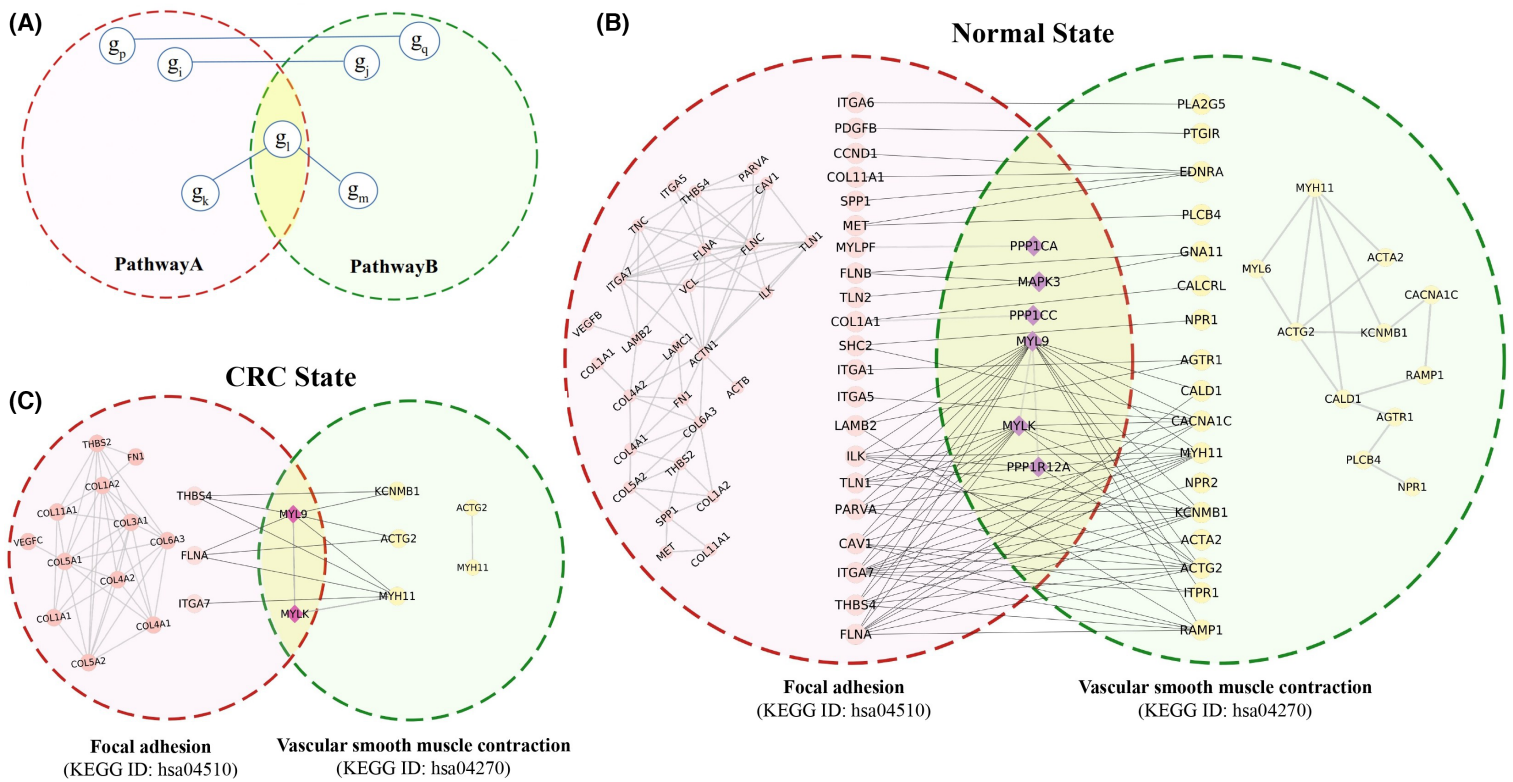
Pathway cross‐talk. (A) Schematic depicts the criteria for constructing the pathway cross‐talk. (B, C) Example of gene co‐expression guided pathway cross‐talk in normal and CRC state respectively.

We considered all possible pairs of selected pathways to construct the pathway cross‐talk networks. The pathway cross‐talk in the network is denoted as G(V, E), nodes (V) represent the set of selected pathways, and edges (E) represent the pathway‐pathway pair guided by the co‐expressed gene pairs. Certain genes are involved in multiple pathways, so a single gene pair might contribute to false positive cross‐talk between pathways. Therefore, we considered the two pathways, for example, PathwayA and PathwayB, to be involved in cross‐talk if they fulfilled at least one of the following criteria as given below and illustrated in Figure 2:

(i) $\left\{g_{i},g_{j}\right\}\;\text{and}\;\left\{g_{p},g_{q}\right\}\in$ co‐expressed gene pairs, where (g_i_ ∧ g_p_ ∈ PathwayA) ∧ (g_i_ ∧ g_p_ ∉ PathwayB) ∧ (g_j_ ∧ g_q_ ∈ PathwayB) ∧ (g_j_ ∧ g_q_ ∉ PathwayA).

(ii) $\left\{g_{k},g_{l}\right\}\;\text{and}\;\left\{g_{l},g_{m}\right\}\in$ co‐expressed gene pairs, where (($g_{k}g_{k}\in$ PathwayA) ∧ ($g_{k}g_{k}\notin$ PathwayB)) ∧ ($g_{l}$ $g_{l}\in$ PathwayA ∧ PathwayB) ∧ (($g_{m}g_{m}\notin$ PathwayA) ∧ ($g_{m}g_{m}\in$ PathwayB)).

This pathway cross‐talk construction methodology is inspired by the review article authored by Azad et al..^1^ An in‐house Python script was used to construct the pathway cross‐talk network. This results in constructing four networks, consisting of pathway cross‐talk and co‐expressed genes involved in inter‐pathway communication in both normal and CRC states. Cytoscape v. 3.10.0^20^ software was used to visualize and analyze the constructed networks in the present study.

### 2.3 Comparative analysis of dependent pathway cross‐talk networks state‐dependent pathway cross‐talk networks

The pathway cross‐talk network of the normal state was compared with the CRC state to understand the importance of the cross‐talk among pathways in the development of CRC. As genes play crucial roles in the cross‐talk among pathways at the molecular level, therefore, the gene pairs connecting two different pathways were also compared and analyzed to identify the crucial genes that might be playing an important role in the pathway cross‐talk required for the CRC progression. An in‐house Python script was used for comparing the path‐ways and gene co‐expression network from normal to CRC state. The gene pairs helping in the construction of pathway cross‐talk networks in the CRC state were further analyzed to identify the key genes involved in pathway cross‐talk as well as in the CRC progression. As each gene pair corresponds to the pathway–pathway pairs, some genes might be involved in regulating more than one pathway. Therefore, we calculated the betweenness centrality for each gene in the co‐expressed gene networks in both states to identify such crucial genes. The betweenness centrality was calculated for each gene using a Cytoscape plugin CytoNCA.^21^ The genes were ranked according to their centrality score, and the top‐ranked genes based on their centrality score were defined as key genes in the present study.

### 2.4 Knowledge‐driven analysis of all the genes involved in pathway cross‐talks

To understand the significance of the gene pairs involved in the pathway cross‐talks, their association with the disease, cancer hallmarks, and genetic alterations analysis was performed. For this, the DisGeNET database^22^ was used to know the disease association of the selected genes, and only the curated association was considered to check the disease association. The genetic alteration was also checked as it plays an important role in the regulation of gene expression as well as in disease development. For this, the 220 samples with mutation data of TCGA, Firehose Legacy of the cBioPortal (https://www.cbioportal.org/) server were analyzed. With the advancement of sequencing data, researchers are also exploring the significance of co‐occurrence or mutual exclusive mutation patterns in cancer as it can provide information about the pathways and their mechanisms in tumor development.^23^ Therefore, we also performed the mutual exclusivity analysis using the cBioPortal server. The association of the selected genes and pathways was checked with the cancer hallmarks using the CHG database (http://www.bio‐bigdata.com/CHG/index.html).^24^

### 2.5 Identification of potential drug candidates

To identify potential therapeutic drugs for CRC and reverse the expression of key genes involved in CRC progression, we explored the L1000CDS ^2^ database (https://maayanlab.cloud/L1000CDS2/#/index). This publicly available database contains over 1 million gene expression profiles of chemically perturbed human cell lines. The database utilizes the characteristic direction (CD) scoring method, which prioritizes genes exhibiting minimal changes but moves together with a large group of other genes.^25^ Genes from identified clusters showing significant expression changes (log2FC) from normal to CRC states across three datasets were selected for this analysis.

### 2.6 Calculation of dysregulated score for genes

Gene co‐expression networks tell the potential functional association between the genes, but they do not provide information on regulatory relationships such as which gene regulates which gene activities. Therefore, we also analyzed the gene regulatory network (GRN) integrated with gene expression data. As interactions among genes are context‐specific, a change in their interaction can lead to disease development. Therefore, we calculated the dysregulation score for each gene based on its change in interaction strength with other genes from the normal state to the CRC state by integrating the gene expression information with the GRN. The directed gene interactions were sourced from the study conducted by Huo et al.^26^ and includes interactions from KEGG, Reactome, Panther, CellMap, and NCI Pathway Interaction Databases. The gene interactions containing the predicted interaction were excluded from the study.

Huo et al.^26^ proposed the method to assess the interaction strength between the genes using their relative expression values and calculated the dysregulation score for each gene by considering all the dysregulated strength of gene i to all its downstream gene. The feedback loop, which involves the regulation of upstream and downstream signaling molecules, plays a critical role in cancer progression.^27^ Therefore, we considered the dysregulated strength of all its interacting genes, including both upstream and downstream interactions. For this, first, we calculated the regulation strength of each gene pair connected in the gene regulatory in both the normal and CRC states separately as.

(1) $$r_{\mathrm{ij}}=\log\frac{E_{g_{i}}}{E_{g_{j}}}$$

Here, r_ij_ represents the regulation strength for the gene i and j connected in the gene regulatory network, $E_{g_{i}}$ and $E_{g_{j}}$ represents the expression value of gene i and gene j. This calculation was performed independently in all three selected datasets. Then, to determine the difference in regulatory strength between the genes from normal to CRC state, we calculated the dysregulation strength (DS) for each gene pair as shown in Equation 2.

(2) $${\mathrm{ds}}_{\mathrm{ij}}=\left|{\bar{r}}_{\mathrm{ij}}^{D}-{\bar{r}}_{\mathrm{ij}}^{N}\;\right|$$

Here, ds_ij_ represents the dysregulation strength (DS) for the N gene pair i and j, ${\bar{r}}_{\mathrm{ij}}^{D}$ and ${\bar{r}}_{\mathrm{ij}}^{N}$ represents the average regulation strength in the CRC and normal state, respectively. After this, the dysregulated score for each gene was calculated. The dysregulated score of all the genes with significant dysregulation strength was calculated by taking the sum of all the dysregulated strength to all its interacting genes.

(3) $$\;d_{i}=\sum_{j=1}^{n_{i}}{\mathrm{ds}}_{\mathrm{ij}}$$

Here, d_i_ represents the dysregulation score of gene i, n_i_ is the number of first neighbors of gene i (interacting genes) in the gene regulatory network. The dysregulated score was calculated for each dataset separately, and to compare the dysregulation strength of genes among all three datasets, the dysregulated score was converted to *Z*‐score = ((dysregulation score − average of all dysregulation score)/standard deviation of all dysregulation score).

### 2.7 Prognostic Analysis of the selected key genes and cross‐validation

To understand the significance of the identified key genes in the progression of the CRC, we performed the survival analysis by using Kaplan–Meier curves in the cBioPortal (https://www.cbioportal.org/).^28^ We also performed the survival analysis for the group of genes that have maintained interactions in both the normal and CRC states. The prognostic significance of these selected groups of genes was assessed at the transcriptomic level using independent gene expression datasets (TCGA RNA‐Seq, GSE28722, and GSE41258). The details of these datasets are provided in Table 1. Cox proportional hazards regression analysis was used to explore the relationship between gene expression and the survival of CRC patients. This analysis was conducted using the SurvExpress (http://victortrevino.bioinformatics.mx:8080/Biomatec/SurvivaX.jsp) web server validation tool.^29^ Risk scores for all CRC samples were calculated using the equation:

(4) $$\text{Risk Score}=\sum_{i=1}^{N}{\mathrm{Exp}}_{i}*\beta_{i}$$

where, Exp_i_ and β_i_ are the expression levels of gene i in a particular patient sample and coefficients of gene i in the multivariate Cox regression analysis. Then, the CRC samples were categorized into low‐risk and high‐risk groups based on the median value of the risk score. The prognostic performance of these gene groups was evaluated by generating Kaplan–Meier plots and assessing the statistical significance using a log‐rank test with a significance *p*‐value < 0.05.

## 3 RESULTS

### 3.1 State dependent KEGG pathway cross‐talks network construction

Pathway cross‐talk networks were systematically constructed based on the criteria mentioned in the material and methods sections (2.2). The example of a pathway cross‐talk network guided by a state‐dependent gene co‐expression network was illustrated in Figure 2B,C. This figure illustrated the pathway cross‐talk between focal adhesion (FA, KEGG ID: hsa04510) and vascular smooth muscle contraction (VSMC, KEGG ID: hsa04270) pathways for both normal and CRC states. Research has also reported the role of the focal adhesion pathway in smooth muscle cell contractility, which is aligning with our analysis.^30^ The figure also showed the co‐expressed genes involved in intra‐pathway cross‐talk. A comparison between inter‐pathway cross‐talk in normal and CRC states reveals alterations in the interactions of many genes during the transition from normal to CRC state. This highlights the significance of cross‐talk at both the pathway and gene levels in maintaining normal cellular function and its role in CRC development. The gene pairs involved in the pathway cross‐talks were considered potential key genes in this study.

**FIGURE 3:**
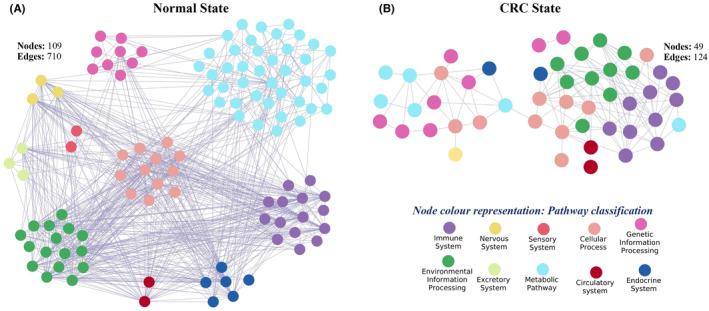
Pathway cross‐talk networks in (A) normal state and (B) CRC state. Pathway classification as per the KEGG database.

In normal state, the pathway cross‐talk network consists of 109 pathways with 710 interactions (Figure 3A). The corresponding gene co‐expression involved in the pathway cross‐talk is termed as PathGeNet (Pathway cross‐talk interface gene co‐expression network) in this study. In the normal state, the PathGeNet consists of 744 genes with 2024 edges (Figure S1). At the gene level, we observed a well‐connected component consisting of 567 genes and many discrete groups of genes. The key genes were also identified by analyzing the PathGeNet, and the top 10 genes based on the betweenness score include PLNA, ITGA7, MYL9, GNA11, FEN1, FLNC, CHP2, HSD11B2, CDK1, and MYLK.

**FIGURE 4:**
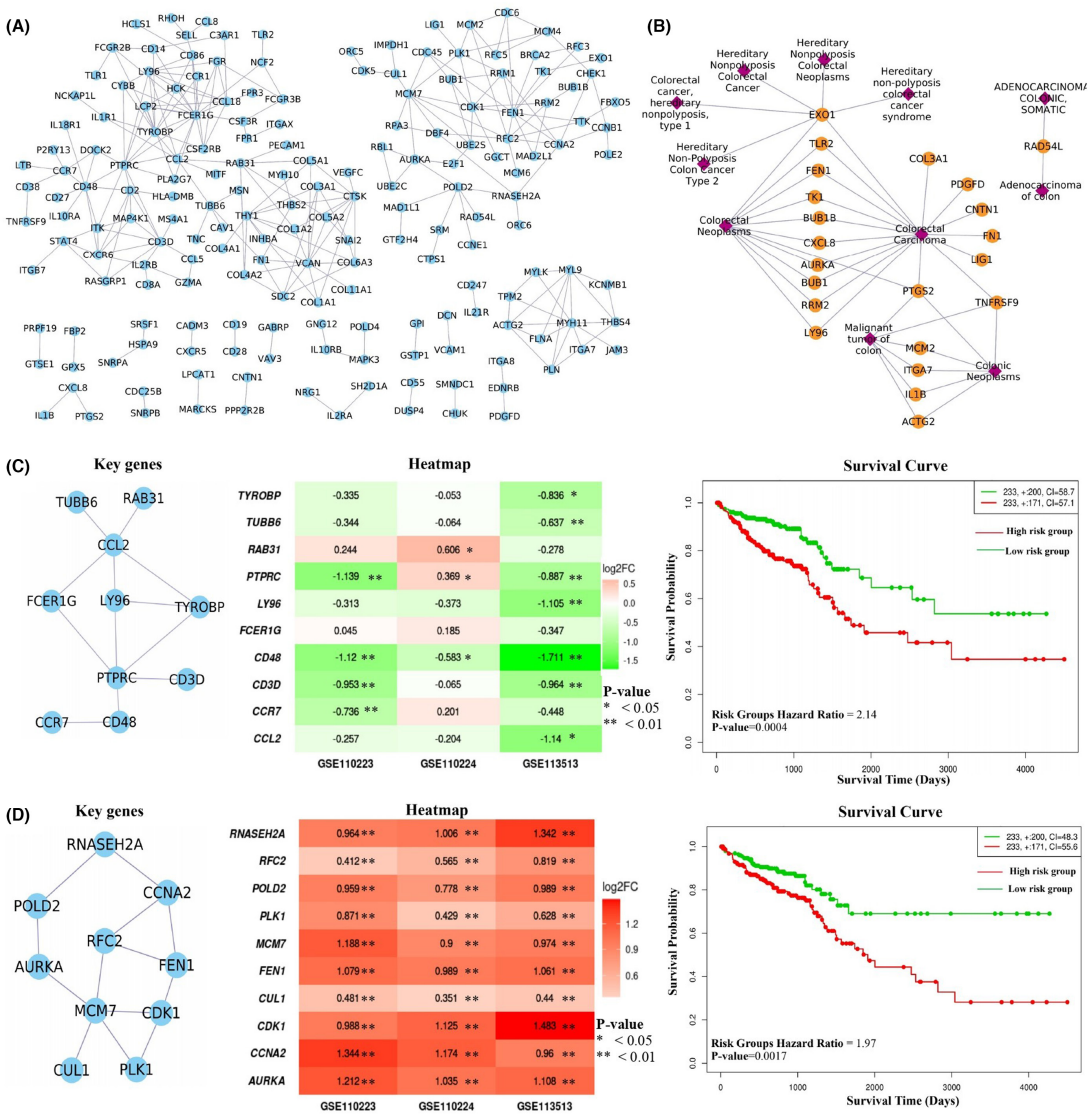
Analysis of pathway cross‐talk in the CRC state. (A) Network of co‐expressed genes involved in pathway cross‐talk. (B) Association of genes with the CRC based on the DisGeNET curated database. (C, D) Identified key genes, heatmap representing the change in their expression (log2FC) pattern and survival analysis from the ClusterK1 and ClusterK2 respectively.

In the CRC state, the pathway cross‐talk networks were composed of 124 interactions among 49 pathways (Figure 3B), and PathGeNet consisted of 273 edges among 182 genes (Figure 4A and Figure S2). At the pathway level, we observed that the gap junction (KEGG ID: hsa04540) pathway acts as a connecting link between two clustered of pathways. At the gene level, we observed two distinct largest connected components (LCCs) of genes in the PathGeNet, namely, *LCC 1* and *LCC 2*. *LCC 1* consists of 81 genes, and the genes of *LCC 1* belong to the immune system and cell communication‐related pathways, whereas 48 genes comprise *LCC 2* and mainly belong to the cell cycle. The topology‐based analysis of these LCCs led us to identify 10 key genes CCL2, CCR7, CD3D, CD48, FCER1G, LY96, PTPRC, RAB31, TUBB6, and TYROBP in *ClusterK1* and 10 key genes AURKA, CCNA2, CDK1, CUL1, FEN1, MCM7, PLK1, POLD2, RFC2, and RNASEH2A in the *ClusterK2*. All the identified key genes were found to be interconnected as shown in Figure 4C,D. At the gene expression level, we observed a variation in the fold change (log2FC) of the key genes belonging to *ClusterK1* in all three datasets, whereas genes from *ClusterK2* were found to be up‐regulated in all three datasets (Figure 4C,D). The consistent expression pattern (log2FC) was observed for these groups of genes in the three independent datasets (Figure S3). Among the identified key genes from the normal state, only two genes, ITGA7 and MYH10, were found to be involved in the pathway cross‐talk in the CRC state. The identified key genes from different clusters were observed to be well interconnected; therefore, their combined prognostic potential was assessed, revealing a significant prognostic power (Figure 4C,D). In all the analyzed independent datasets, the survival probability was observed to be significantly decreased over time in the high‐risk group compared to the low‐risk group (Figure S4). The Wilcoxon rank‐sum analysis revealed that deceased individuals had significantly higher risk scores than those who were alive, indicating an association between high‐risk scores and mortality risk in CRC patients (Figure S4). An analysis of risk score distributions over time revealed that the high‐risk group had a greater number of CRC patients who experienced death compared to the low‐risk group (Figure S4). This suggests the potential ability of these key gene clusters as prognostic biomarkers for CRC patients.

### 3.2 State dependent comparative analysis of interaction at KEGG pathway and gene level

**FIGURE 5:**
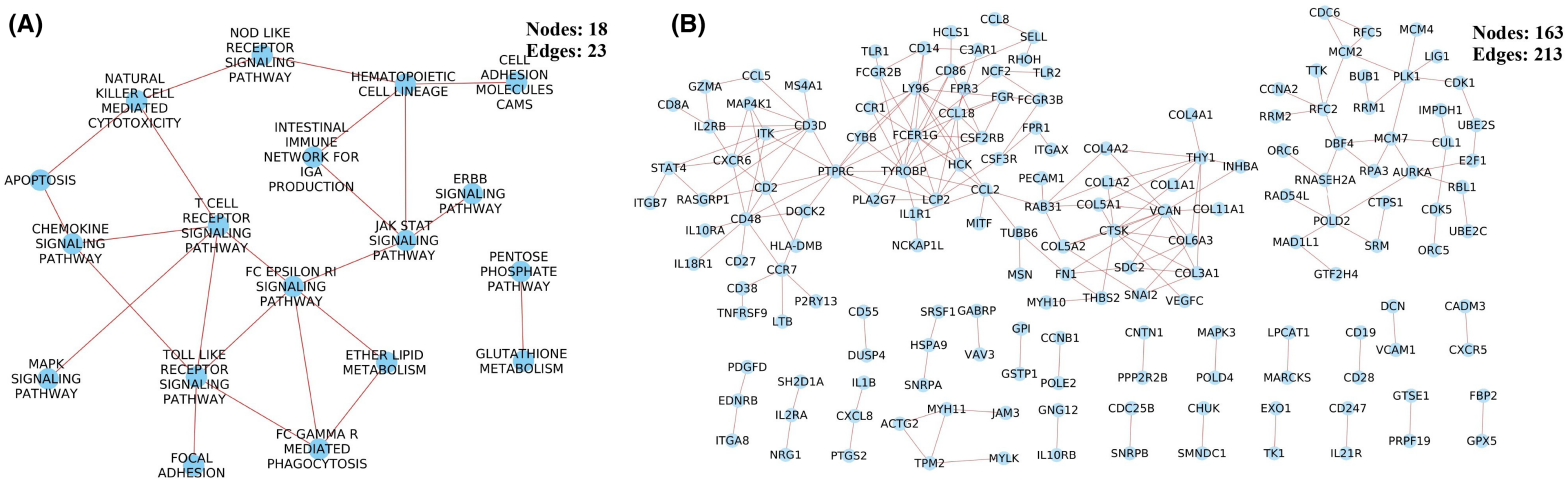
Interactions among (A) pathways and (B) their corresponding co‐expression gene pairs observed only in the CRC state compared to the normal state.

Network comparison was made at both the pathway and gene level to identify the pathways and genes that undergo significant changes in cross‐talk during the transition from a normal to a CRC state. In the normal state, 637 interactions were identified among 109 pathways and 744 genes with 1961 interactions specifically involved in pathway cross‐talk unique to the normal state when compared to the CRC state (Figure S5). In the CRC state, we observed 23 cross‐talks among 18 pathways and 213 interactions among 163 genes at the gene level only in the CRC state compared to the normal state, as shown in Figure 5. These cross‐talks were mainly observed among the pathways related to the immune system, signaling pathways such as chemokine, MAPK, and JAK–STAT signaling pathway.

**FIGURE 6:**
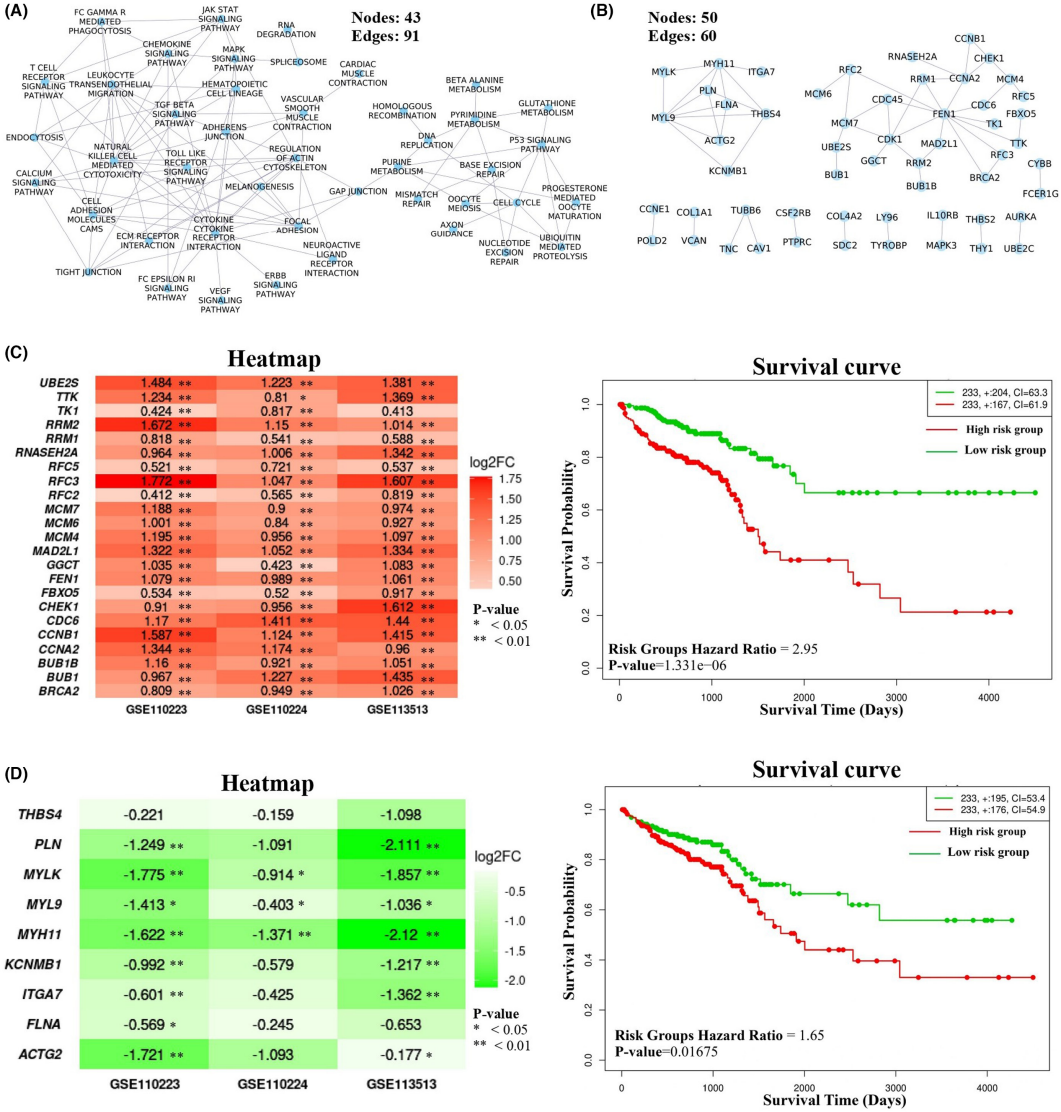
Overlapping interactions between normal and CRC state at (A) pathway level and (B) co‐expressed gene involved in pathway cross‐talk. (C, D) Heatmap and survival curve plot represent the change in the expression (log2FC) of genes and their respective prognostic significance in the CRC for the genes with conserved co‐expression.

In the comparison of pathway cross‐talk networks, it was observed that 101 cross‐talks among 44 pathways overlapped in both states, as shown in Figure 6A. At the gene level, the overlapping interactions among 55 genes were observed in both states (Figure 6B). Among these 55 genes, 25 genes were found clustered together and mainly associated with the cell cycle‐related pathways, and the expression of all these genes was found to be up‐regulated in the CRC state (Figure 6C). Likewise, nine genes related to cell–cell communication pathways were observed in one cluster, and all were down‐regulated in the CRC state, as shown in Figure 6D. Survival analysis was also performed on conserved gene clusters, and it was observed that both the clusters could differentiate between patients at higher risk and those at lower risk in all the three independent datasets, as shown in Figure 6 and Figure S4. We called these clusters as *ClusterK3* and *ClusterK4* based on the number of genes, respectively.

### 3.3 Knowledge‐driven analysis of the genes involved in KEGG pathway cross‐talks

**FIGURE 7:**
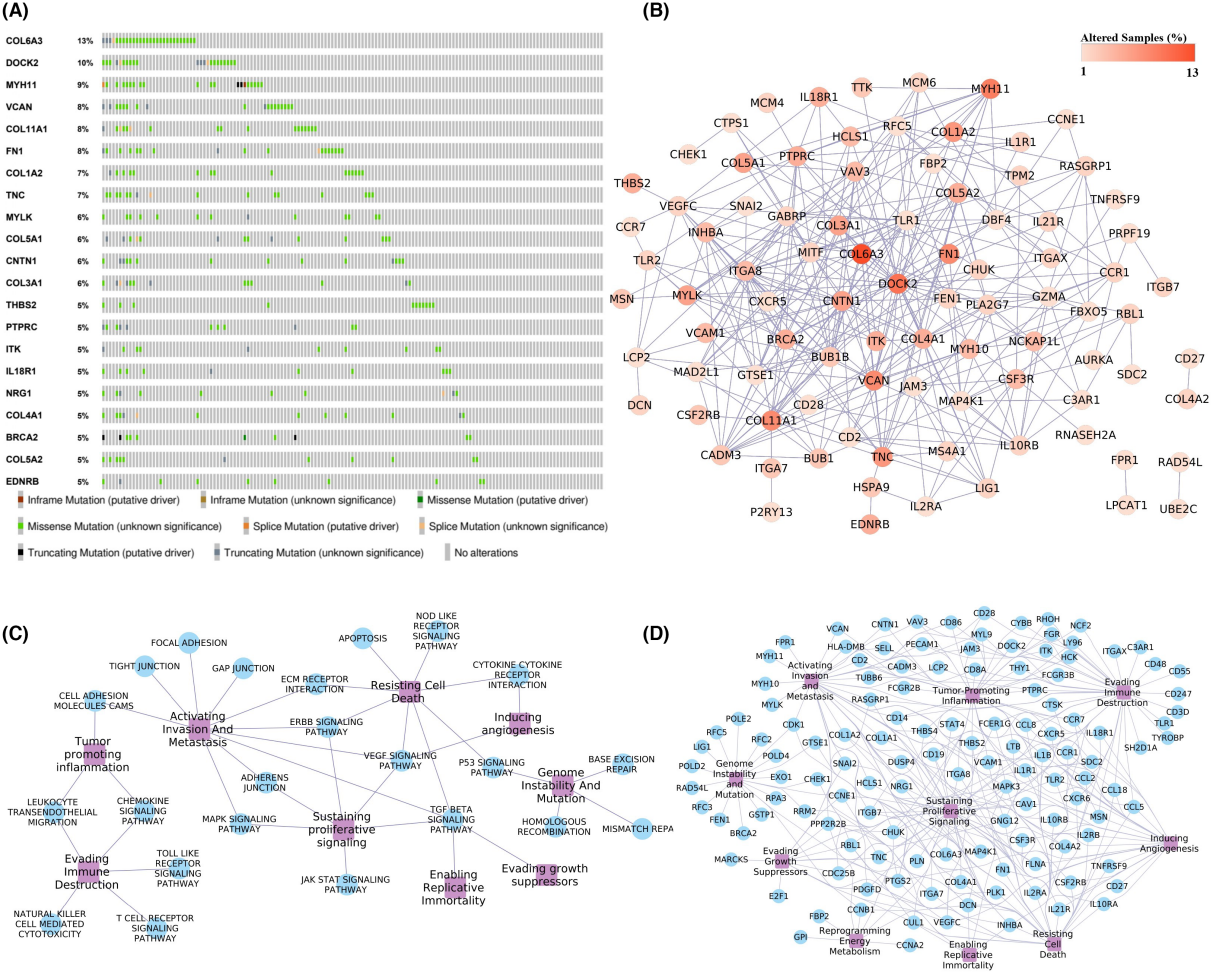
Genetic alteration and cancer hallmarks analysis. (A) Oncoprint showing the distribution of genetic alterations in the CRC. (B) The gene network showing the co‐occurring mutation pattern among genes. The color gradient of nodes represents the percentage of the genetic alteration of the genes in samples. (C) Association of pathways and (D) genes with the cancer hallmarks. The purple color node represents the cancer hallmarks.

The disease‐gene association analysis revealed that among 182 genes in pathway cross‐talk in the CRC state, 95 genes were associated with at least one type of cancer (Figure S6), and 22 genes were found to be associated with the CRC (Figure 4B). Four key genes CCL2, CCR7, LY96, and PTPRC, from *ClusterK1* and five genes AURKA, CDK1, CCNA2, FEN1, and PLK1 from *ClusterK2*, were associated with at least one type of cancer. LY96, FEN1, and AURKA key genes were known CRC‐associated genes. The genetic alterations of these genes in 220 CRC patient samples were assessed using the cBioPortal, and we found out that, except for 20 genes, all the genes were showing alterations. Figure 7A shows the genes' genetic alteration observed in at least 5% of the CRC patients. The COL6A3 gene was found to be altered in 13% of the samples, whereas the DOCK2 gene was altered in 10% of the samples, as shown in Figure 7A. The co‐occurring mutation patterns were observed among the 328 gene pairs, and the DOCK2 gene showed co‐occurrence with 32 genes, including COL6A3 gene (Figure 7B). The co‐occurring mutation pair is reported to generally activate different complementary biological pathways, resulting in different cancer characteristics with a few exceptions in the tumorigenesis.^23^ Among the identified key genes, five genes, AURKA, CCR7, FEN1, PTPRC, and RNASEH2A, showed co‐occurring mutations with other genes. Cancer hallmarks analysis results revealed that 23 pathways and 128 genes were associated with at least one type of cancer hallmark, as shown in Figure 7C,D. These results highlight the significance of genes participating in pathway cross‐talk in the CRC.

### 3.4 State dependent Reactome pathway cross‐talks network analysis

The state‐dependent pathway cross‐talk network was constructed for the 1475 Reactome pathways using specified selection criteria and network construction methods outlined in the materials and methods section (2.2). A consistent rewiring pattern in pathway cross‐talk was observed in Reactome and KEGG pathways analyses. In the normal state, 1,57,071 cross‐talks were identified among 1347 pathways whereas 53,100 cross‐talks were observed among 909 pathways in the CRC state. While some pathways lost connectivity in CRC, others maintained, and some formed new interactions (Figure S7). The immune‐related pathways, followed by signaling pathways such as chemokine signaling, were observed to be the most rewired pathways (Figure S7).

**FIGURE 8:**
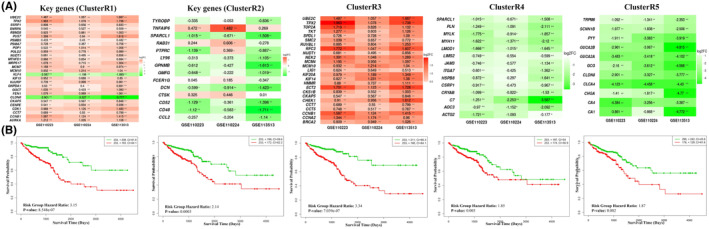
Reactome pathway cross‐talk analysis. (A) Heatmap showing the change in gene expression (log2FC) for the identified gene clusters from the Reactome PathGeNet analysis and (B) Survival curve plot representing the prognostic significance of these gene clusters in CRC using TCGA RNA‐seq datasets.

At the gene level, PathGeNet analysis in the CRC state identified two distinct largest connected components (LCCs) of genes, which include LCC1, consisting of 557 genes, and LCC2, comprising 265 genes. Analyzing the network topology of these LCCs revealed 28 key genes in LCC1 and 14 key genes in LCC2, named as *ClusterR1* and *ClusterR2*, respectively. Survival analysis of these key genes clustered showed their prognostic significance in distinguishing low‐risk from high‐risk groups (Figures S10, S11, and S12), indicating their potential impact on CRC progression (Figure 8A,B). These findings highlight the importance of these genes in pathway cross‐talk and their role in CRC prognosis.

The comparison of PathGeNet among normal and CRC states identified the three different conserved sub‐networks: sub‐network1, consisting of 116 genes; sub‐network2, comprised of 67 genes; and sub‐network 3, which included 11 genes. The sub‐network1 and sub‐network2 were further analyzed with Cytoscape plugin jActiveModule^31^ to identify the smaller clusters of genes showing significant changes in gene their expression patterns, and they were termed as *ClusterR3* comprising 25 genes and *ClusterR4* consisting of 14 genes. The sub‐network3 with 11 genes showed a significant change in the log2FC from normal to CRC; therefore, it was considered as ClusterR5. These all three clusters showed their potential impact on CRC progression by distinguishing between low‐ and high‐risk groups (Figures S11 and S12).

A comparative analysis of PathGeNet in the CRC state showed a partial overlap of key genes as well as clustered genes such as MCM7, AURKA, FEN1, CDK1, POLD2, FCER1G, PT‐PRC, CCL2, RAB31, CD48, TYROBP, and LY96 identified in both the KEGG and Reactome analysis. The independent analysis of Reactome identified a unique gene cluster, *ClusterR5*, which was not present in the KEGG analysis (Figures S8 and S9). The *ClusterR5* mainly involved in the ion transport channels, cell–cell communication and digestion and absorption‐related Reactome pathways. CA4 gene of the *ClusterR5* is a known diagnostic and prognostic biomarker for CRC.^32^, ^33^ The analysis of disease‐gene associations revealed that 10 genes (FEN1, GCG, ACTG2, LY96, TPX2, AURKA, GPNMB, ITGA7, LIG1, and SCNN1B) part of the identified clustered from Reactome analysis (Figure S12). This highlights the importance of conducting individual pathway analyses to capture specific gene clusters that might be missed in the combined analyses.

### 3.5 Identification of potential drug candidates

All the identified clustered showing significant association with CRC progression were searched against the L1000CDS^2^ database, resulting in 36 unique small molecules with a score >0.5 showing reverse changes in gene expression (Table S1). Seven of these molecules, including Palbociclib (an approved drug for breast cancer treatment), were found to reverse gene expression in HT29 (human colorectal adenocarcinoma) cell lines (Table 2). The chemical structure of these seven drug candidates was shown in Figures S17 and S18.

**TABLE 2:** List of potential candidate drugs as therapeutics for CRC (HT29 cell line).

Drug Name (PubChem ID)^a^	Target information			Indication^b^
	Overlap Score^a^	Up‐regulated^c^	Down‐regulated^c^	
DL‐PDMP (16219895)	0.7241	AURKA, BUB1, CCNA2, CCNB1, CDC6, CDK1, CHEK1, FBXO5, FEN1, MAD2L1, MCM4, MCM6, MCM7, PLK1, RFC2, RFC3, RFC5, RNASEH2A, RRM1, RRM2, UBE2S	NA^d^	Investigative stage PMID: 31900415
GDC‐0980 (25254071)	0.7241	AURKA, BUB1, CCNA2, CDC6, CDK1, CHEK1, FEN1, GGCT, MAD2L1, MCM4, MCM6, MCM7, PLK1, POLD2, RFC2, RFC3, RFC5, RNASEH2A, RRM1, RRM2, UBE2S	NA^d^	Non‐hodgkin lymphoma: Phase2 Solid tumor/cancer: Phase1
CAM‐9‐027‐3 (49849912)	0.69	AURKA, BUB1, CCNA2, CCNB1, CDC6, CDK1, CHEK1, FEN1, MAD2L1, MCM4, MCM6, MCM7, PLK1, RFC2, RFC3, RFC5, RNASEH2A, RRM1, RRM2, UBE2S	NA^d^	Pre‐clinical stage (CHEMBL1873309)
PF 750 (25154868)	0.69	AURKA, BUB1, CCNA2, CCNB1, CDC6, CDK1, CHEK1, FBXO5, FEN1, MAD2L1, MCM4, MCM6, MCM7, PLK1, RFC2, RFC3, RFC5, RRM1, RRM2, UBE2S	NA^d^	Investigative stage
WH‐4‐025 (73707529)	0.69	AURKA, BUB1, CCNA2, CDC6, CDK1, CHEK1, FEN1, MAD2L1, MCM4, MCM6, MCM7, PLK1, POLD2, RFC2, RFC3, RFC5, RNASEH2A, RRM1, RRM2, UBE2S	NA^d^	NA^d^
Foretinib (42642645)	0.69	AURKA, BUB1, BUB1B, CCNA2, CDC6, CDK1, CHEK1, FEN1, MAD2L1, MCM4, MCM6, MCM7, PLK1, POLD2, RFC2, RFC3, RFC5, RNASEH2A, RRM2	MYH11	Breast cancer: Phase 2 Gastric adenocarcinoma: Phase 2 Renal cell carcinoma: Phase 2 Squamous head and neck cell carcinoma: Phase 2 Solid tumor/cancer: Phase 1
Palbociclib (5330286)	0.69	AURKA, BUB1, BUB1B, CCNA2, CDC6, CDK1, CHEK1, FBXO5, FEN1, MAD2L1, MCM4, MCM6, MCM7, PLK1, RFC2, RFC3, RFC5, RRM1, RRM2, UBE2S	NA^d^	Breast cancer: Approved solid tumor/cancer: Phase 2

^a^ L1000CDS2 database; Overlap score: overlap between the input DE genes and the signature DE genes divided by the effective input.

^b^ TTD: therapeutic target database.

^c^ DEGs with *p*‐value < 0.01 and average log2FC genes in all the three datasets ≥ |0.5|.

^d^ Not applicable.

### 3.6 Gene regulatory network analysis

**FIGURE 9:**
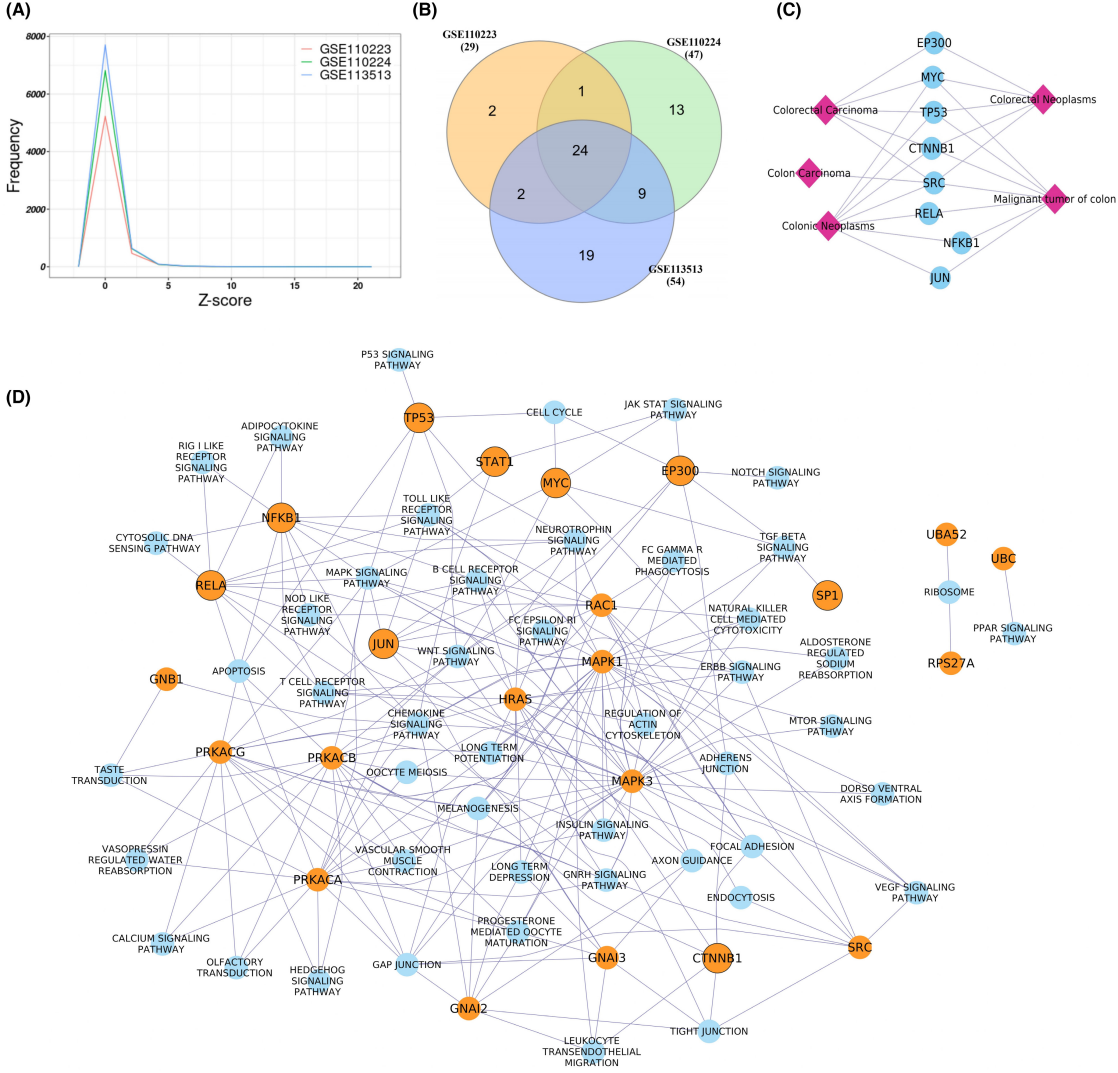
Analysis of dysregulated genes. (A) Distribution of dysregulated score (*Z*‐score) in all three datasets. (B) A Venn diagram representing the common dysregulated genes with *Z*‐score ≥5 among all three datasets. (C) Association of identified dysregulated genes with the CRC. (D) Association of identified dysregulated genes with the KEGG Pathways. The nodes with orange colors represent the dysregulated genes, and the nodes with black boundaries represent the TFs identified as dysregulated genes.

The dysregulated score was calculated for all the genes using directed gene regulatory networks in all three datasets. The 24 genes were found to be common among all the three datasets with *Z*‐score ≥5 (Figure 9A,B) and considered as key dysregulated genes in the CRC. These dysregulated scores were calculated in the three independent datasets comprising normal and CRC samples, revealing that all 24 identified dysregulated genes overlapped with gene *Z*‐scores ≥4, except for two genes (PRKACG and HRAS) in the RNA‐seq datasets (Figures S13 and S14). This analysis highlights the potentially significant role of these dysregulated genes in CRC development. Excluding the pathway associated with disease, pathway enrichment analysis results revealed that these 24 genes were mainly enriched in Relaxin signaling (hsa:04926), Chemokine signaling (hsa:4062), thyroid hormone signaling (hsa:04915) (Table 3). Among 24 genes, nine genes were reported as transcription factors CTNNB1, EP300, JUN, MYC, NFKB1, RELA, SP1, STAT1, and TP53 based on the TTRUST database (https://www.grnpedia.org/trrust/) of gene regulation. Disease gene association analysis revealed that all 24 key dysregulated genes except GNAI3, PRKACG, RPS27A, and UBB were associated with at least one type of cancer (Figure S14), whereas eight genes (MYC, TP53, CTNNB1, JUN, RELA, EP300, SRC, and NFKB1) were associated with the CRC as shown in Figure S15. Among all the identified 24 dysregulated genes, only the CTNNB1 gene showed a poor survival probability with the altered gene expression, as shown in Figure S15. We also checked the CTNNB1 prognostic significance in other cancers, and we found that CTNNB1 showed a significant survival probability (*p*‐value < 0.05) in the nearby organs of CRC, such as the bladder, liver, and pancreas, but did not show any significant results in the distant organs such as breast and lung tissue (Figure S15).

**TABLE 3:** Top 10 KEGG enriched Pathways of the identified key dysregulated genes.

KEGG ID	Pathway name	Gene count	*p*‐Value	Genes
hsa04926	Relaxin signaling pathway	13	1.08E‐16	JUN,SRC,GNAI3,RELA,NFKB1,GNAI2,PRKACG,GNB1,MAPK1,PRKACA,HRAS,PRKACB,MAPK3
hsa04062	Chemokine signaling pathway	14	2.59E‐16	STAT1,SRC,GNAI3,RELA,NFKB1,GNAI2,PRKACG,GNB1,MAPK1,RAC1,PRKACA,HRAS,PRKACB,MAPK3
hsa04919	Thyroid hormone signaling pathway	12	3.71E‐15	STAT1,SRC,PRKACG,MYC,EP300,MAPK1,CTNNB1,PRKACA,HRAS,TP53,PRKACB,MAPK3
hsa04915	Estrogen signaling pathway	11	9.12E‐13	JUN,SRC,SP1,PRKACG,GNAI3,MAPK1,PRKACA,HRAS,PRKACB,GNAI2,MAPK3
hsa04916	Melanogenesis	10	2.43E‐12	PRKACG,GNAI3,EP300,MAPK1,CTNNB1,PRKACA,HRAS,PRKACB,GNAI2,MAPK3
hsa04024	cAMP signaling pathway	12	3.75E‐12	JUN,PRKACG,GNAI3,EP300,MAPK1,RAC1,PRKACA,PRKACB,RELA,NFKB1,GNAI2,MAPK3
hsa04540	Gap junction	9	4.26E‐11	SRC,PRKACG,GNAI3,MAPK1,PRKACA,HRAS,PRKACB,GNAI2,MAPK3
hsa04010	MAPK signaling pathway	12	9.18E‐11	JUN,PRKACG,MYC,MAPK1,RAC1,PRKACA,HRAS,TP53,PRKACB,RELA,NFKB1,MAPK3
hsa04921	Oxytocin signaling pathway	10	1.14E‐10	JUN,SRC,PRKACG,GNAI3,MAPK1,PRKACA,HRAS,PRKACB,GNAI2,MAPK3

## 4 DISCUSSION

In the present study, we integrated the gene co‐expression network with the pathways information to understand the significance of pathway cross‐talks in the CRC progression. Since pathway cross‐talks are condition‐dependent,^2^ we constructed and analyzed pathway cross‐talk networks and their corresponding gene co‐expression networks (PathGeNet) separately for normal and CRC states. Furthermore, we integrated the gene regulatory network with gene expression data to identify the dysregulated genes and assess their impact on pathway cross‐talks in CRC.

In our study, we conducted separate analyses of the KEGG and Reactome pathways to explore a wide range of gene sets and curated pathways. This dual analysis provided enhanced insights into the molecular mechanisms involved in the CRC progression. A consistent rewiring patterns in pathway cross‐talk were observed in both KEGG and Reactome pathways analyses. While certain pathways lost connectivity in CRC, others maintained, and some formed new interactions. The immune‐related pathways, cell cycle‐related pathways, and signaling pathways such as chemokine signaling were observed to be the most rewired pathways in both the analysis highlighting their significance in the progression of CRC. The immune system plays a significant role in both preventing and progressing CRC due to its pro‐tumor and anti‐tumor activities.^34^ In our analysis, we identified the pathways related to immune systems were involved in cross‐talks with each other and with other pathways, such as TGF‐β signaling. Our analysis found that among immune‐related pathways, the Natural Killer (NK) cell‐mediated cytotoxicity pathway and the T cell receptor (TCR) signaling pathway were the most common pathways facilitating the communication both within the immune system and with other pathways. NK and T cells are essential immune system components and play a crucial role in the immunosurveillance and elimination of cancer cells. Studies have reported the pivotal role of NK and T cell‐mediated cytotoxicity in eliminating cancer cells and its potential in immunotherapy. It is reported that the NK and T cells share some cytotoxic mechanisms, such as the release of perforin and granzymes, which can be effective against cancer cells.^35^, ^36^

In our analysis, we also observed the cross‐talk between the NK‐cell‐mediated cytotoxicity and TCR signaling pathways in the CRC state, which was not observed in the normal state. The genes FCER1G, TYROBP, and PTPRC, associated with NK cell‐mediated cytotoxicity and TCR signaling pathways, were identified as key genes in both KEGG and Reactome analyses. This highlights the significance of these identified key genes in pathway cross‐talk between immune pathways and others. In the CRC state, our analysis showed that the TCR signaling pathway had increased interactions with other immune pathways and the MAPK signaling pathways compared to the normal state. PTPRC gene was found to be mediating the cross‐talk among immune pathways as well as with other pathways. PTPRC (protein tyrosine phosphatase receptor type C), also known as CD45, is an essential transmembrane glycoprotein present on the surface of immune cells. It plays an essential role in regulating B and T‐cell receptor signaling. Within TCR pathway, PTPRC regulates the activity of FYN and LCK kinases, members of the Src family required to activate TCR signaling. Studies have reported that the JAK family of proteins act as a substrate for PTPRC and play a regulatory role in the cytokine and chemokine signaling pathway.^37^, ^38^, ^39^, ^40^

We also observed the cross‐talk of TCR signaling pathway with JAK–STAT, chemokine, and cytokine–cytokine receptor interaction pathways. We identified PTPRC as one of the connecting link between these cross‐talk in the CRC state. Cytokines and chemokines play a crucial role in inflammation, animportant element of the tumor microenvironment (TME) and one of the cancer hallmarks. Cytokines are small proteins that play a key role in activating immune cells during infections or tissue damage. Chemokines are one of the key players in the metastasis of the CRC.^41^, ^42^ Genetic alteration analysis revealed mutations in the PTPRC gene in 5% of CRC patient samples. Interestingly, mutual exclusivity analysis revealed a co‐occurring mutation pattern of PTPRC with genes IL1R1, IL18R1, and VEGFC from the cytokine pathway, and DOCK2 gene from the chemokine signaling pathway. DOCK2 (Dedicator of Cytokinesis 2) is reported to play a crucial role in the regulation and activation of T cells and NK cells. Notably, our findings align with previous reports identifying DOCK2 as the most frequently mutated gene in CRC.^43^ These observations suggest the important role of PTPRC in pathway cross‐talks in the CRC state. Our analysis also found the cross‐talk of TCR signaling pathway with the MAPK signaling pathways in the CRC state. MAP4K1, also known as Hematopoietic Progenitor Kinase 1 (HPK1), is a member of the MAPK signaling pathway and was found to co‐expressed with CD3D and ITK genes, which are integral parts of the TCR signaling pathway in the present analysis. Studies have reported that MAP4K1 is a crucial negative feedback regulator in TCR signaling.^44^ This observation highlights the role of the TCR signaling pathway and its cross‐talk with other pathways in the CRC.

Resistance to apoptosis is one of the characteristics of cancer. In our analysis, we observed a new cross‐talk between apoptosis and chemokine signaling, as well as NK cell‐mediated cytotoxicity and TCR signaling in the CRC state, compared to the normal state. Studies have reported the apoptosis resistance to the chemokine signaling and NK cell‐mediated cytotoxicity pathways in cancer.^35^, ^45^ CCL2, a chemokine, is a key gene identified in our study through KEGG and Reactome analyses found to mediate the cross‐talk of the chemokine signaling pathway with apoptosis and other pathways. Studies have reported the role of CCL2 in the Epithelial‐Mesenchymal Transition (EMT) and metastasis in CRC.^46^

The cell–cell and cell‐ECM (extracellular matrix) interactions are essential for maintaining homeostasis and tissue integrity as well as playing a significant role in the migration and proliferation of CRC.^47^ The ECM, a complex network comprising collagens, laminins, elastin, glycoproteins, and proteoglycans, provides structural support to cells and regulates essential cellular functions such as growth, differentiation, and migration. Notably, ECM is one of the major components of the TME.^48^ The contact point of cell‐ECM is termed focal adhesion, essential for maintaining tissue integrity.^49^ Tight junctions maintain cell polarity, while the actin cytoskeleton provides structural support to cells, maintaining cell shape, movement, and regulating cell‐extracellular communication. These pathways have been reported to play an important role in the EMT process. The interactions of adhesion protein with the actin are required to maintain the integrity of the epithelial cell.^50^, ^51^ The cell adhesion molecules (CAMs) involved in cell–cell and cell‐matrix interactions and alterations in the expression of these adhesion molecules impact immune responses and help in the progression and migration of CRC.^52^, ^53^ Our analysis also observed a conserved interaction among these pathways and with immune‐related pathways such as NK cell‐mediated cytotoxicity and TCR signaling pathways in both normal and CRC state. The CD2 gene of the CAMs pathway is an adhesion molecule that plays an important role in the formation of immunological synapses between the T cells and Antigen‐presenting cells (APCs) upon stable cell–cell interaction and activation of T cells and NK cells. It also affects the T cell receptor‐peptide‐loaded MHC complex required for the T cell activation.^54^ Our study also observed the cross‐talk among the CAMs pathway and TCR and NK cell‐mediated cytotoxicity pathways in both the normal and CRC states, which suggests the significance of cross‐talk among these pathways in both the normal and CRC states. Furthermore, our study identified the transcription factor SNAI2, a member of adherence junctions, involved in cross‐talk with the focal adhesion pathway. At the gene level, SNAI2 was found to co‐express with COL3A1, COL5A2, and COL6A3, key members of the focal adhesion pathway. SNAI2, a critical regulator of EMT, has been implicated in promoting EMT in gastric cancer by upregulating COL5A2 expression.^55^ These findings highlight the essential role of intercellular communication in maintaining normal cellular functions and driving CRC progression.

Besides these, we also observed the conserved interaction between the endocytosis and immune pathways, including TCR signaling and CAM pathways, in normal and CRC states. Studies have highlighted the impact of endocytosis on the TME, particularly through its effects on CAMs and immune cells. RAB31 (Ras‐related protein Rab‐31), a member of the RAB GTPases protein family, is pivotal in regulating vesicle endocytic trafficking by regulating GTP and GDP binding. Increased expression of RAB31 has been associated with CRC progression, with studies indicating its involvement in cross‐talk with signaling pathways like HGF‐MET signaling.^56^, ^57^ In our analysis, RAB31 and IL2RB of the endocytosis pathways were found to mediate the cross‐talks with other pathways in the CRC state. This finding suggests the potential role of endocytosis in CRC development.

The cell communication‐related pathways were also identified in cross‐talk with the calcium signaling and vascular smooth muscle cell contraction pathways in both the normal and CRC state. At the gene level, conserved interactions were observed among ACTG2, MYLK, MYL9, KCNMB1, PLN, THBS4, ITGA7, MYH11, and FLNA genes in both states. Among these genes, ACTG2, MYLK, MYL9, and MYH11 act downstream and KCNMB1 upstream of the vascular smooth muscle cells pathway. PLN and MYLK genes of the calcium signaling pathways were seen in cross‐talk with other pathways, including the vascular smooth muscle cell contraction pathway. Calcium signaling plays critical roles in both normal and pathological cellular activities and remodeling of TME.^58^, ^59^ Studies have reported that in TME, cancer cells lack smooth muscle cells to promote the spread of cancer from primary sites to distant locations. A study conducted by Li et al.^60^ demonstrated that CRC cells induce apoptosis in vascular smooth muscle cells to promote the metastasis of CRC. A review article by Ribeiro‐Silva et al.^30^ highlighted the role of focal adhesion signaling in vascular smooth muscle cell contractions and also discussed the role of the actin cytoskeleton in smooth muscle contractions. Our analysis also observed cross‐talks among vascular smooth muscle contraction pathways with the tight junction, focal adhesion, and regulation of actin cytoskeleton pathway in both the normal and CRC states.

At the expression level, we observed a decrease in the expression of the genes within ClusterK4, ClsuerR4, and ClusterR5 in the CRC state. ITGA7, also known as Integrin Subunit Alpha 7, is a member of the integrin family that functions as cell‐matrix adhesion receptors for transducing signals and modulating a range of cellular processes. Various studies have reported the significance of integrins in the metastasis of various types of cancer.^61^, ^62^ Li et al.^63^ showed that the downregulation of ITGA7 is associated with CRC growth and metastasis. Our study observed that the ITGA7 has maintained its interactions with the MYH11 genes. However, no new interactions of ITGA7 were observed in CRC. The MYH11 is a smooth muscle myosin heavy chain 11 protein involved in muscle contraction and plays a crucial role in the function of smooth muscles. A few studies have reported the role of ITGA7 in regulating vascular smooth muscle cells.^64^, ^65^ These results are consistent with Li et al.^63^ study and suggest the ITGA7 significance in maintaining the normal function of cells. ITGA7 was also identified as the most rewired gene from normal to CRC state in our previous study.^19^ This observation underscores the critical role of ITGA7 in maintaining cellular functionality in normal state and in the progression of CRC. A study by Tan et al.^66^ in their study showed that the low expression of both the MYL9 and MYLK were associated with non‐small cell lung cancer (NSCLC). THBS4 (Thrombospondin‐4) member of the ECM receptor interaction, focal adhesion and TGF‐β signaling pathway was reported as a tumor suppressor gene in the CRC.^67^ The survival analysis of these group of genes MYH11, MYL9, PLN, THBS4, ITGA7, ACTG2, FLNA, KCNMB1, and MYLK also showed their significant prognostic power, which suggests that these groups of genes could be explored as prognostic biomarkers for the CRC. The JAM3 gene was also observed only in the pathway cross‐talk between the vascular smooth muscle contraction and tight junction in the CRC state. JAM3 is a Junctional Adhesion Molecules (JAMs) subfamily member that regulates tight junctions in epithelial and endothelial cells. In CRC, JAM3 has been reported as a tumor suppressor gene and plays a crucial role in the growth and migration of tumor cells.^68^ These findings indicate that the altered expression, as well the interactions among the genes of cell–cell communication pathway crucial for maintaining cell integrity and shape, and alteration in these pathways contributes to the progression of CRC.

The p53 signaling pathway was found in cross‐talk with mainly metabolic‐related pathways such as glutathione metabolism, purine and pyrimidine metabolism in the CRC state. P53 also plays an important role in regulating metabolic pathways, including glutathione, purine, and pyrimidine synthesis, consistent with our study.^69^ The p53 signaling pathway, governed by the tumor suppressor p53, exerts crucial functions in regulating the cell cycle, apoptosis, and DNA repair to prevent cancer development. Dysregulation or mutations in p53 have been implicated in various cancers, including CRC. Notably, our study identified p53 as a key dysregulated gene, emphasizing its pivotal role in CRC progression. Cancer is a result of abnormal cell cycle activity. Our study observed cross‐talk of cell cycle pathways with the genetic information processing pathways, including DNA replication and nucleotide excision repair (NER) pathway in the CRC state. DNA replication is part of the cell cycle, and NER is an important part of DNA repair systems. Studies have reported the association of these pathways with the risk and prognosis of CRC.^70^, ^71^ Among the identified key genes from ClusterK2, ClusterK3, ClusterR1, and ClsuterR3, genes such as MCM7, FEN1, POLD2, CCNA2, RFC2, CCNB1, RNASEH2A, CDK1, CUL1, PLK1 were found to be involved in the cross‐talk between the cell growth pathways and other pathways. These clustered of genes suggested a potential synergistic effect in the CRC progression. Chemical perturbation analysis of the gene expression profiles identifies the seven candidate drug molecules with reversed expression in these genes upon treatment at the cell line level. One of these drugs, Palbociclib, has already been approved for treating breast cancer (https://idrblab.net/ttd/data/drug/details/d00uzr) and is currently being tested in combination with other drugs in Phase II clinical trials for CRC treatment (https://clinicaltrials.gov/search?term=Palbociclib&cond=CRC). This suggests that the candidate drug molecules identified in our study could be further explored for repurposing in CRC treatment, potentially leading to more effective and targeted therapeutic strategies.

**FIGURE 10:**
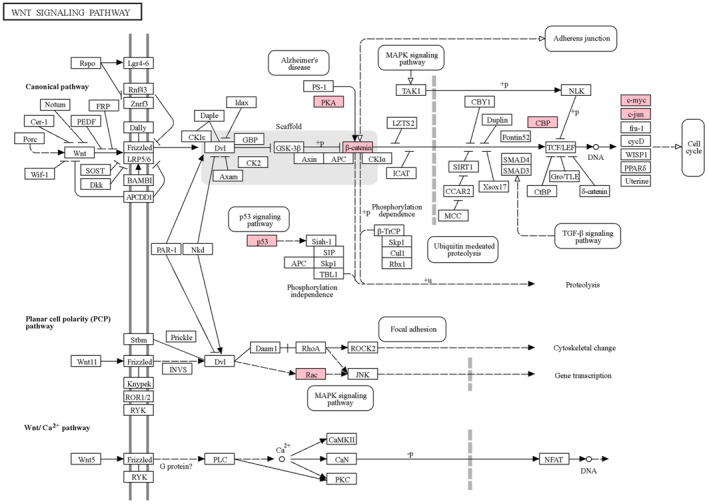
Wnt signaling (KEGG ID: hsa04310) KEGG Pathway represents the identified dysregulated genes in the present study. Light pink color boxes represent dysregulated genes.

TFs play a crucial role in biological systems by regulating gene expression and downstream signaling pathways.^72^ Among the identified dysregulated genes, nine genes were found to be the TFs. Among these, NFKB1, RELA, STAT1, and SP1 are the tumor oncogene, and TP53 is a tumor suppressor gene in CRC. NFKB1 and RELA are part of the NF‐KB complex, the key regulator of tumorigenesis and inflammation in CRC.^73^ These two TFs were the common TFs among the immune‐related pathways, MAPK signaling and apoptosis pathway, as shown in Figure 9D. JUN, also known as C‐Jun, is a part of the AP‐1 transcription factor family and common TFs among different pathways, including the immune‐related pathway, focal adhesion, MAPK, and Wnt signaling pathways. In CRC, the expression of JUN was reported to be upregulated and play an important role in the cancer progression by making a complex with the TCF4 and β‐catenin (CTNNB1) transcription factors.^73^, ^74^ CTNNB1 was also identified as a key dysregulated gene in our analysis. It is a part of the Wnt signaling pathway, which regulates various biochemical processes such as cancer Cell Proliferation, metabolism, inflammation and immunization, heterogeneity and metastasis. The Wnt signaling pathway is considered as an important signaling pathway involved in the initiation of CRC^75^, ^76^ and among 24 key dysregulated genes, nine genes CTNNB1, EP300, JUN, MYC, RAC1, PRKACA, PRKACB, PRKACG, and TP53 were involved in Wnt signaling pathway as shown in Figure 10. The survival analysis results showed the significance of CTNNB1 in the progression of CRC. We found that CTNNB1 inhibits the EP300 gene in the gene regulatory network, which is also a key dysregulated gene in our analysis. EP300 (E1A Binding Protein P300), also known as P300 is an acetyltransferase and acts as a transcription co‐activator, identified as one of the key dysregulated genes in our analysis. Gene regulatory network analysis also revealed that EP300 regulates or makes a complex with 17 other identified key dysregulated genes in this study. In recent years, studies have reported the role of EP300 as a tumor‐promoting factor in various cancers, including CRC stomach cancer, and play an important role in the regulation of various biological processes such as activating oncogene, regulation of immune function, and tumor cell growth.^77^ These findings suggest the significance of the identified dysregulated gene in the CRC.

**FIGURE 11:**
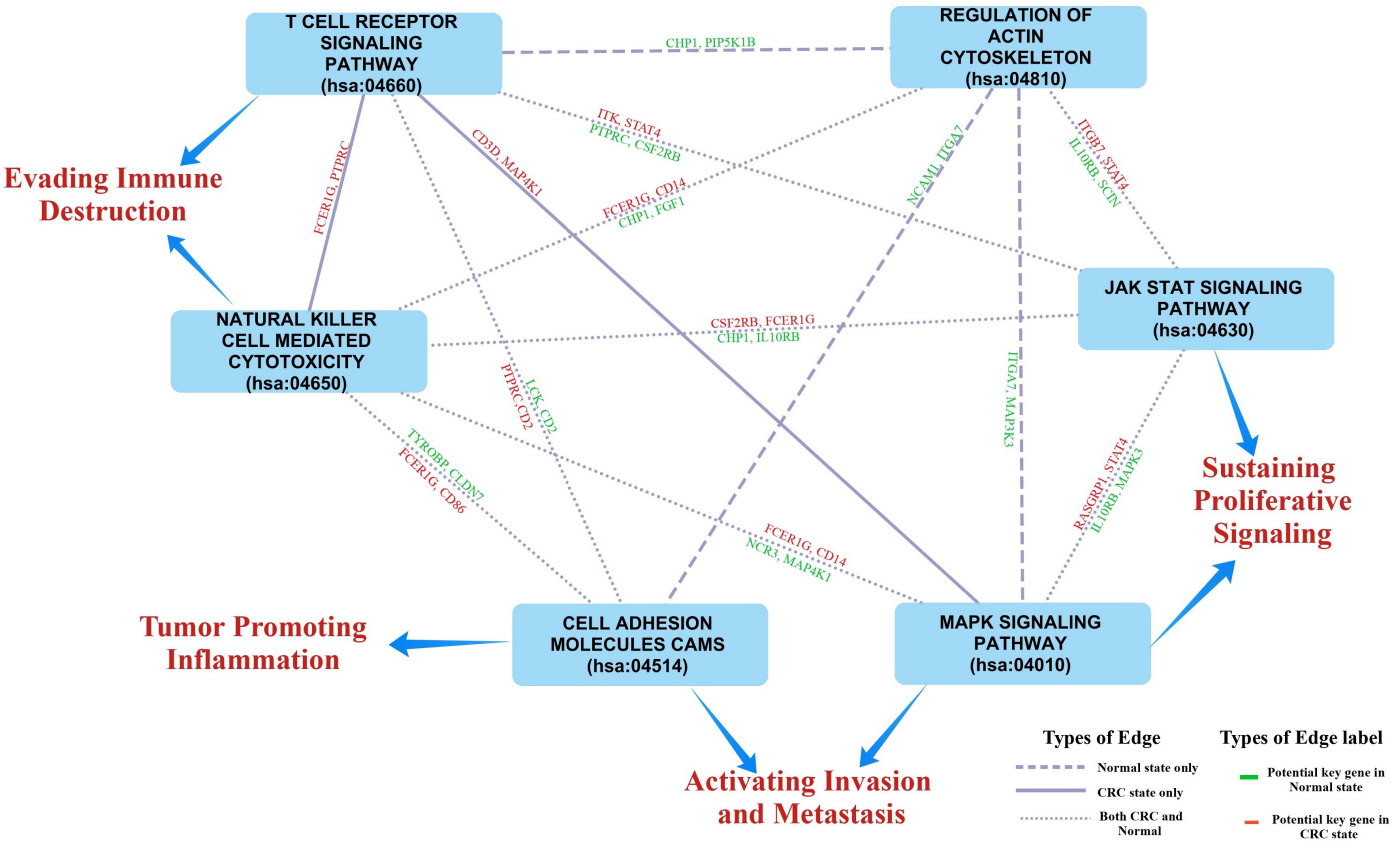
Rewired KEGG pathway cross‐talk network from the normal to the CRC state. The edge label in the network represents the key potential gene involved in the pathway cross‐talk.

This study also explored the role of these identified key dysregulated genes in the observed pathway cross‐talk in both the normal and CRC states. Four genes, namely, GNAI2, MAPK3, PRKACB, SRC, and STAT1, were found to be involved in the pathway cross‐talk in the normal state only, while MAPK3 genes were observed to be part of pathway cross‐talk in both the normal and CRC state. This observation suggests the significance of the rewiring of the interaction at both pathways and gene levels in the development of the CRC. Figure 11 shows the example of the rewired pathway cross‐talk in this study with the selected key potential genes observed in this pathway cross‐talk. The information of these selected genes is provided in Table 4. While this analysis sheds light on pathway cross‐talk in CRC progression, it is important to acknowledge the limitations. The identification of key genes was data‐driven, relying on publicly available datasets. Future research should aim to confirm these findings through experimental validation to enhance the reliability and clinical relevance of the observed pathway cross‐talk findings.

**TABLE 4:** Description of the selected genes involved in the rewired KEGG pathway cross‐talks.

Gene Symbol	KEGG ID	Description
CD14	hsa:929	Monocyte differentiation antigen CD14
CD2	hsa:914	T‐cell surface antigen CD2
CD3D	hsa:915	CD3 delta subunit of T‐cell receptor complex
CD86	hsa:942	T‐lymphocyte activation antigen CD86
CHP2	hsa:63928	Calcineurin like EF‐hand protein 2
CLDN7	hsa:1366	Claudin 7
CSF2RB	hsa:1439	Cytokine receptor common subunit beta
FCER1G	hsa:2207	Fc epsilon receptor Ig
IL10RB	hsa:3588	Interleukin 10 receptor subunit beta
ITGA7	hsa:3679	Integrin subunit alpha 7
ITGB7	hsa:3695	Integrin subunit beta 7
ITK	hsa:3702	IL2 inducible T cell kinase
JAM3	hsa:83700	Junctional adhesion molecule 3
LCK	hsa:3932	LCK proto‐oncogene, Src family tyrosine kinase
MAP4K1	hsa:11184	Mitogen‐activated protein kinase 1
MAPK3	hsa:5595	Mitogen‐activated protein kinase 3
MYH10	hsa:4628	Myosin heavy chain 10
NCR3	hsa:259197	Natural cytotoxicity triggering receptor 3
PIP5K1B	hsa:8395	Phosphatidylinositol‐4‐phosphate 5‐kinase type 1 beta
PTPRC	hsa:5788	Protein tyrosine phosphatase receptor type C
RASGRP1	hsa:10125	RAS guanyl releasing protein 1
SCIN	hsa:85477	Scinderin
STAT4	hsa:6775	Signal transducer and activator of transcription 4
TYROBP	hsa:7305	Transmembrane immune signaling adaptor TYROBP

MicroRNAs (miRNAs), which regulate gene expression at post‐transcriptional levels, were explored to find out the interconnection between the gene involved in the pathway cross‐talk in CRC and key dysregulated genes using g:Profiler.^78^, ^79^ Three microRNAs (miRNAs), namely hsa‐miR‐193b‐3p, hsa‐miR‐29b‐3p, and hsa‐miR‐146a‐5p, were identified as common regulatory molecules regulating the gene involved in the pathway cross‐talk in the CRC state as well as identified key dysregulated genes (Figure S16). The hsa‐mir‐146a‐5p and hsa‐mir‐193b‐3p among these miRNAs have associations with various cancer types, including gastric cancer.

## 5 CONCLUSIONS

The present work focuses on understanding the significance of pathway cross‐talks in CRC by considering the condition‐specific characteristics of pathway interactions. We integrated the gene co‐expression networks with the domain knowledge to explore the cross‐talks in both normal and CRC states separately. Through integrated network analysis, this study identifies immune‐related pathways and those associated with cell communication and signaling pathways as crucial in CRC progression. PTPRC and ITGA7 were identified as the crucial genes in the rewired pathway cross‐talk and epithelial‐mesenchymal transition in CRC, respectively. The identified key dysregulated genes like CTNNB1, EP300, JUN, MYC, NFKB1, RELA, SP1, STAT1, and TP53 act as common regulators across various pathways. The survival analysis highlights the prognostic significance of the identified gene cluster, which could be explored as a potential prognostic biomarker for CRC. L1000CDS^2^ analysis identified the seven potential drug candidates DL‐PDMP, GDC‐0980, CAM‐9‐027‐3, PF 750, WH‐4‐025, foretinib, and palbociclib molecules for the genes of in ClusterK2 and ClusterK3 involved in CRC progression. The ability of these drugs to reverse the expression of genes associated with CRC progression suggests as potential candidate molecules for further investigation and may lead to better outcomes in clinical application. Overall, this study enhances our understanding of the molecular mechanisms involved in CRC progression by emphasizing the importance of pathway cross‐talk analysis. The identification of potential prognostic biomarkers and key dysregulated genes provides valuable insights for further research and potential therapeutic interventions in CRC. The analysis of potential drug candidates using L1000CDS2 further expands the scope for targeted treatment strategies in CRC.

## AUTHOR CONTRIBUTIONS

**Mohita Mahajan:** Data curation (lead); formal analysis (lead); methodology (lead); resources (lead); validation (lead); writing – original draft (lead); writing – review and editing (lead). **Angshuman Sarkar:** Formal analysis (supporting); investigation (supporting); supervision (supporting); writing – review and editing (supporting). **Sukanta Mondal:** Conceptualization (lead); data curation (supporting); formal analysis (equal); methodology (lead); project administration (lead); resources (lead); supervision (lead); writing – original draft (lead); writing – review and editing (lead).

## FUNDING INFORMATION

No funding was received for conducting this study.

## CONFLICT OF INTEREST STATEMENT

The authors declare no conflict of interest.

## Supporting information


Table S1.

Figures S1–S18.

## Data Availability

The datasets analyzed during the current study are available in the Gene Expression Omnibus database (GEO, https://www.ncbi.nlm.nih.gov/geo/), MSigDB (Molecular Signature Database, https://www.gsea‐msigdb.org/gsea/msigdb) and L1000CDS 2 database (L1000 Characteristic Direction Signature Search Engine, https://maayanlab.cloud/L1000CDS2/). The code used in our experiment is available at https://github.com/Mohita1304/Pathway‐Cross‐talk‐analysis.git.
