# Supplementary material for: Integrative network analysis of transcriptomics data reveals potential prognostic biomarkers for colorectal cancer
Source: Cancer Med. 2024 Jun 14;13(11):e7391. doi: 10.1002/cam4.7391 (PMC11176588; doi:10.1002/cam4.7391)
Supplement: Supplementary file 1 — Table S1. [file CAM4-13-e7391-s001.docx]

**Table S1.** Association of identified miRNA with cancer, miRCancer database (http://mircancer.ecu.edu/search.Jsp).

| miR ID | Cancer | Profile | PubMed Article |
| --- | --- | --- | --- |
| hsa-mir-146a-5p | bladder cancer | up | Expression Level of Urinary MicroRNA-146a-5p Is Increased in Patients With Bladder Cancer and Decreased in Those After Transurethral Resection. DOI: 10.1016/j.clgc.2016.04.002 |
| hsa-mir-146a-5p | gastric cancer | down | The clinical significance of downregulation of mir-124-3p, mir-146a-5p, mir-155-5p and mir-335-5p in gastric cancer tumorigenesis. DOI: 10.3892/ijo.2014.2415 |
| hsa-mir-146a-5p | hepatocellular carcinoma | down | Down-regulation of miR-146a-5p and its potential targets in hepatocellular carcinoma validated by a TCGA- and GEO-based study. DOI: 10.1002/2211-5463.12198 |
| hsa-mir-146a-5p | non-small cell lung cancer | down | MiR-146a-5p inhibits cell proliferation and cell cycle progression in NSCLC cell lines by targeting CCND1 and CCND2. DOI: 10.18632/oncotarget.11040 |
| hsa-mir-193b-3p | gastric cancer | down | Dysregulation of NCAPG, KNL1, miR-148a-3p, miR-193b-3p, and miR-1179 may contribute to the progression of gastric cancer. DOI: 10.1186/s40659-018-0192-5 |
