# Supplementary material for: Integrative network analysis of transcriptomics data reveals potential prognostic biomarkers for colorectal cancer
Source: Cancer Med. 2024 Jun 14;13(11):e7391. doi: 10.1002/cam4.7391 (PMC11176588; doi:10.1002/cam4.7391)
Supplement: Supplementary file 2 — Figures S1–S18. [file CAM4-13-e7391-s002.pdf]

# Supplementary Information

## Integrative network analysis of transcriptomics data reveal potential prognostic biomarkers for colorectal cancer

Mohita Mahajan, Angshuman Sarkar and Sukanta Mondal*

Department of Biological Sciences,

Birla Institute of Technology and Science, Pilani, K.K. Birla Goa campus

Goa, 403726, India

# Section 1: KEGG pathway cross-talk analyses

Please refer '**Figure 1'** in the main text for the workflow

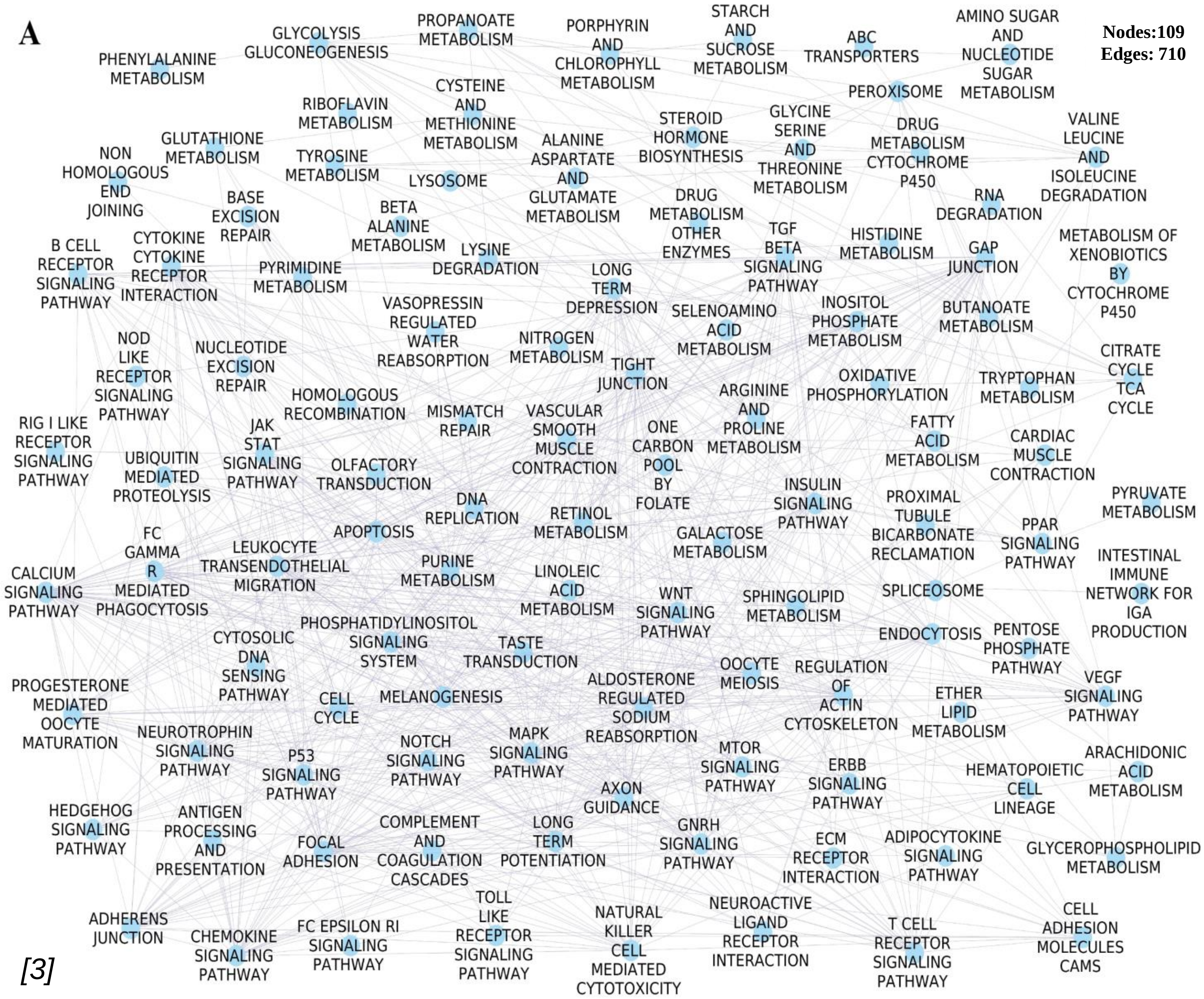


**Figure S1. (A)** Pathway cross-talk network in normal state.

**B**

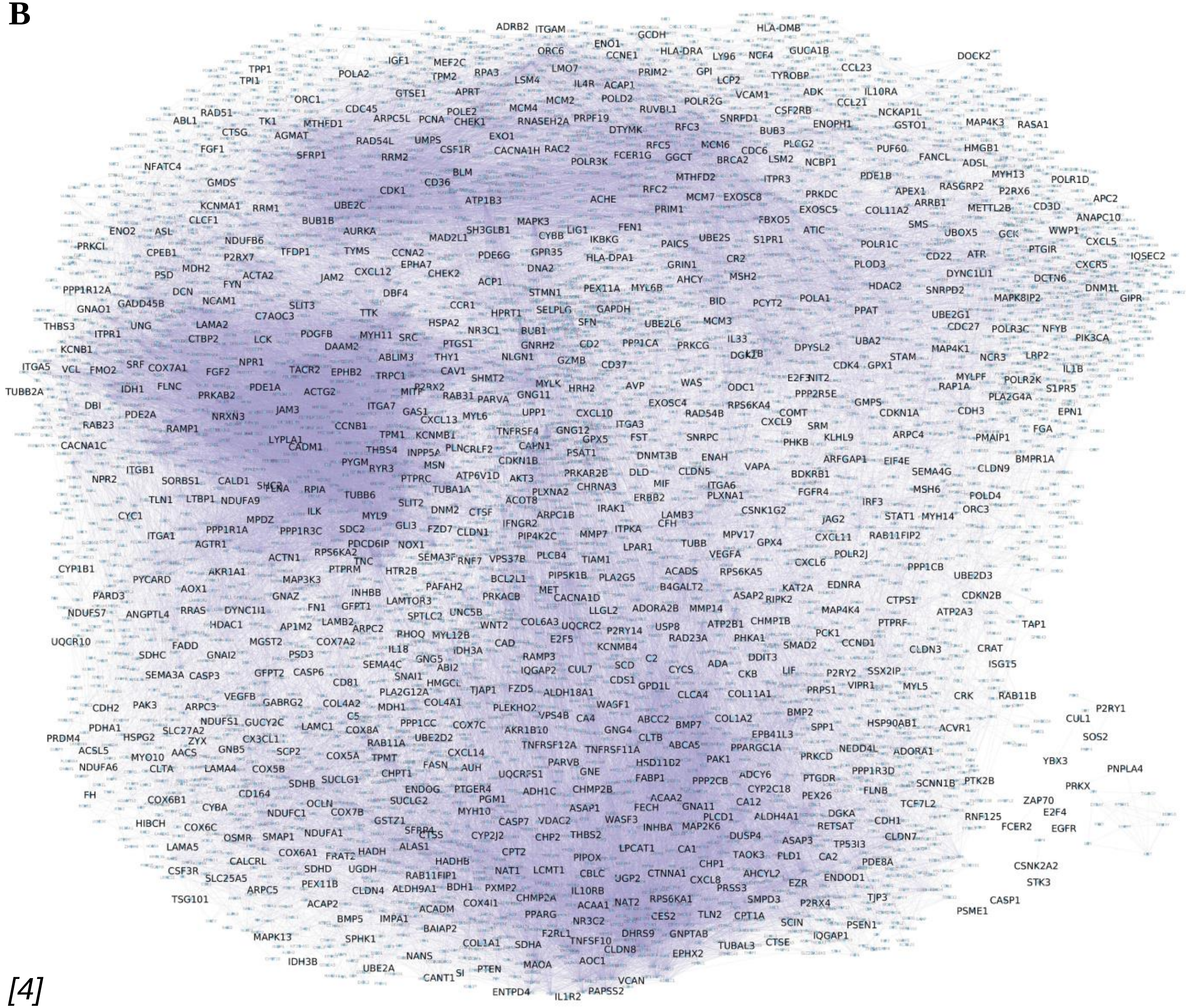

**No. of nodes:** 5259
**No. of nodes in LCC :** 4827
**No. of edges:** 46848
**No. of edges in LCC:** 46585

**Figure S1. (B)** The largest connected component (LCC) of the co-expressed gene in the normal state. The highlighted nodes in the network correspond to genes involved in pathway cross-talk in normal state.

**C**

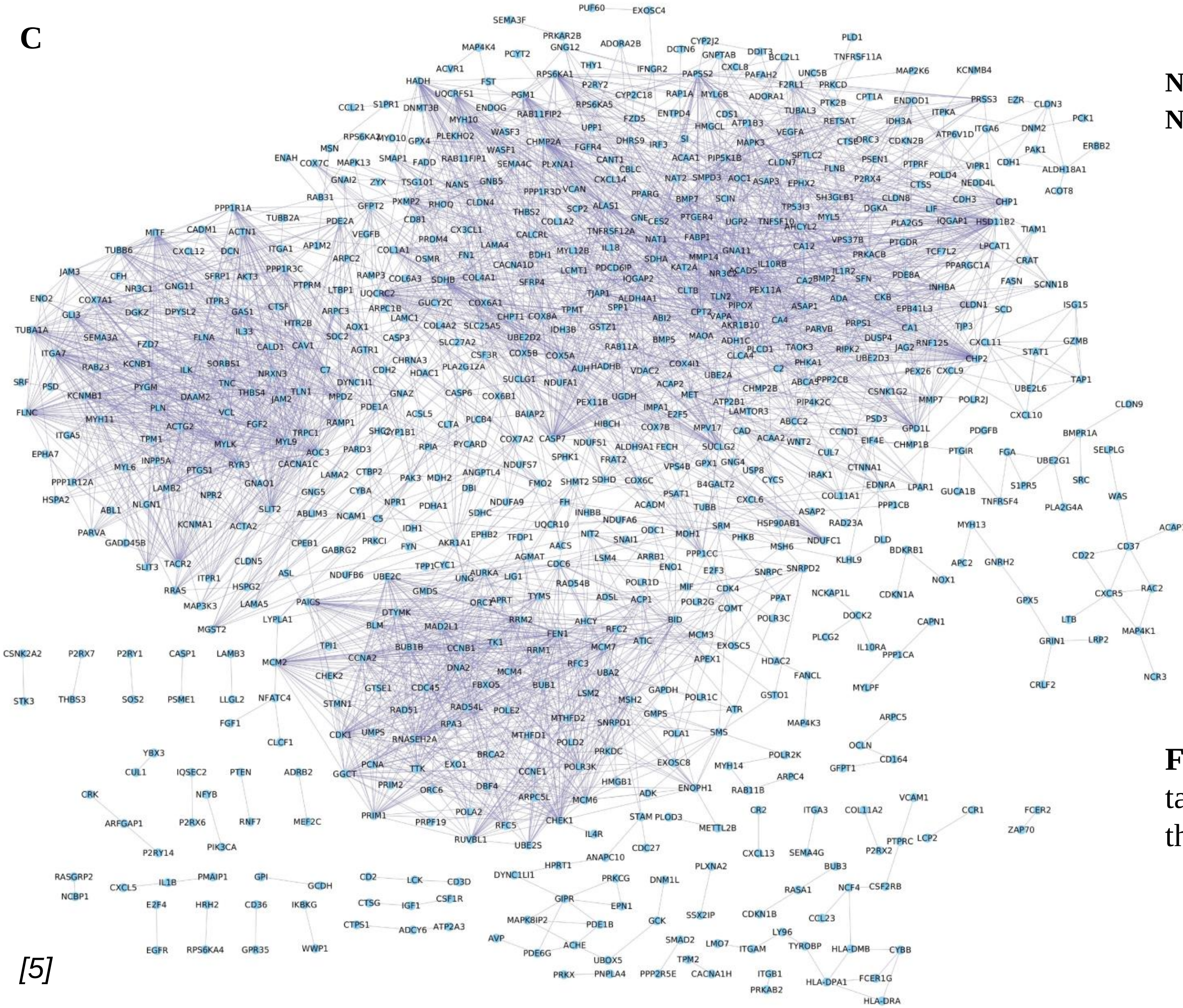

**No of nodes:** 744
**No of edges:** 2024

**Figure S1. (C)** PathGeNet (pathway cross-talk interface gene co-expression network) in the normal state.

**A**

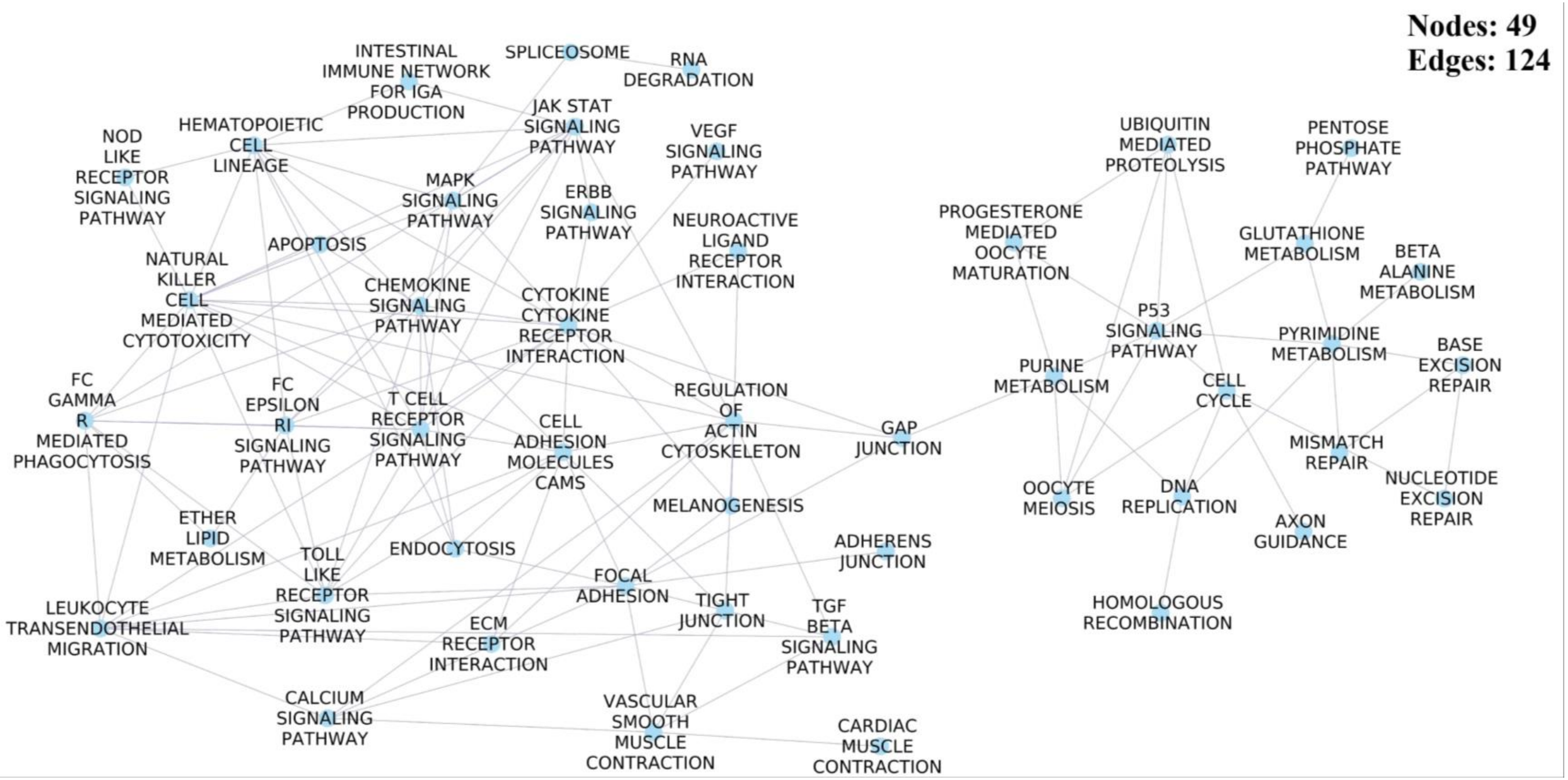


**Figure S2. (A)** Pathway cross-talk in CRC state.

**B**

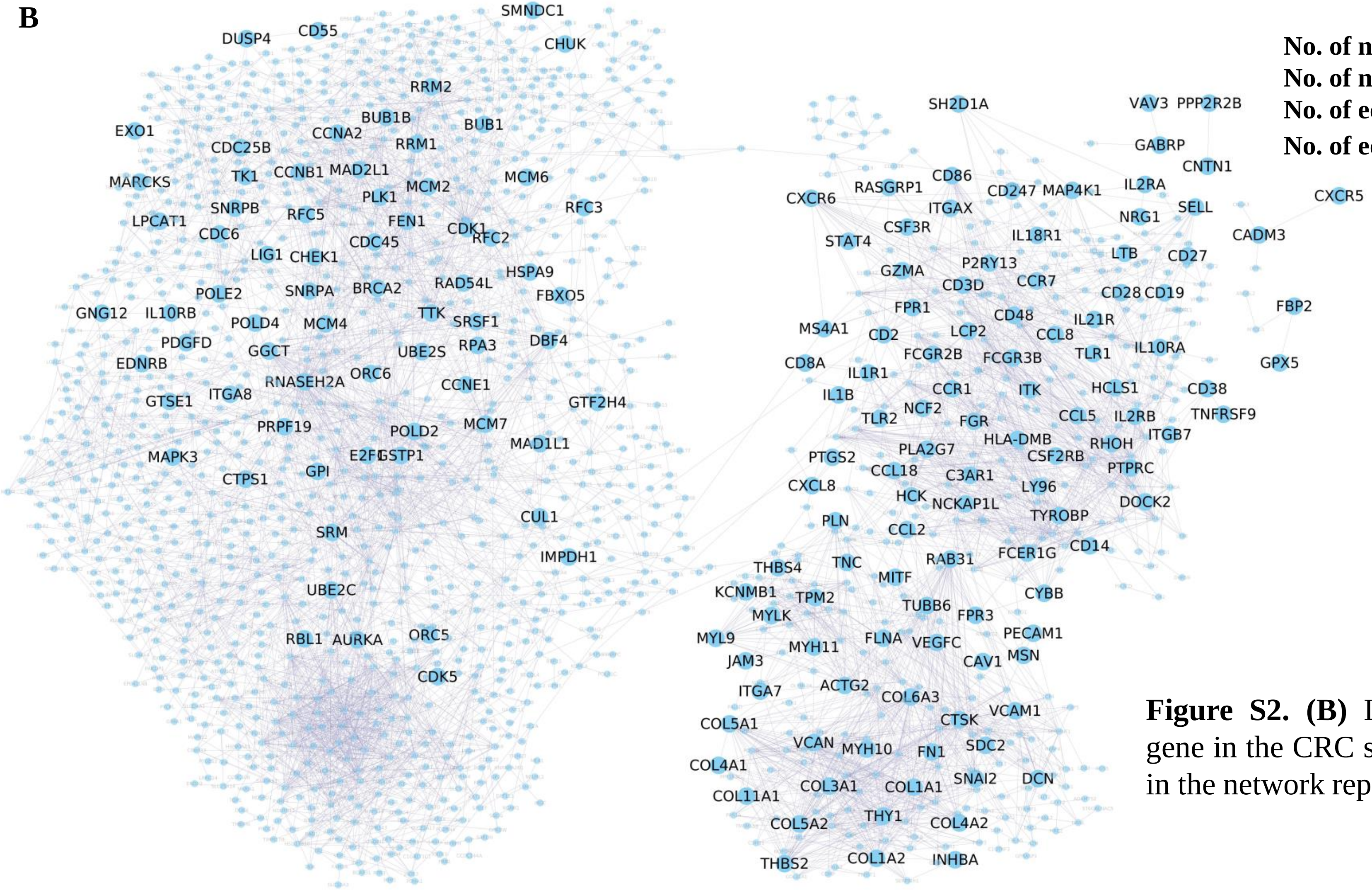


**No. of nodes:** 2210
**No. of nodes in LCC :** 1610
**No. of edges:** 5729
**No. of edges in LCC:** 5349

**Figure S2. (B)** LCC of the co-expressed gene in the CRC state. The highlighted nodes in the network represents the PathGeNet.

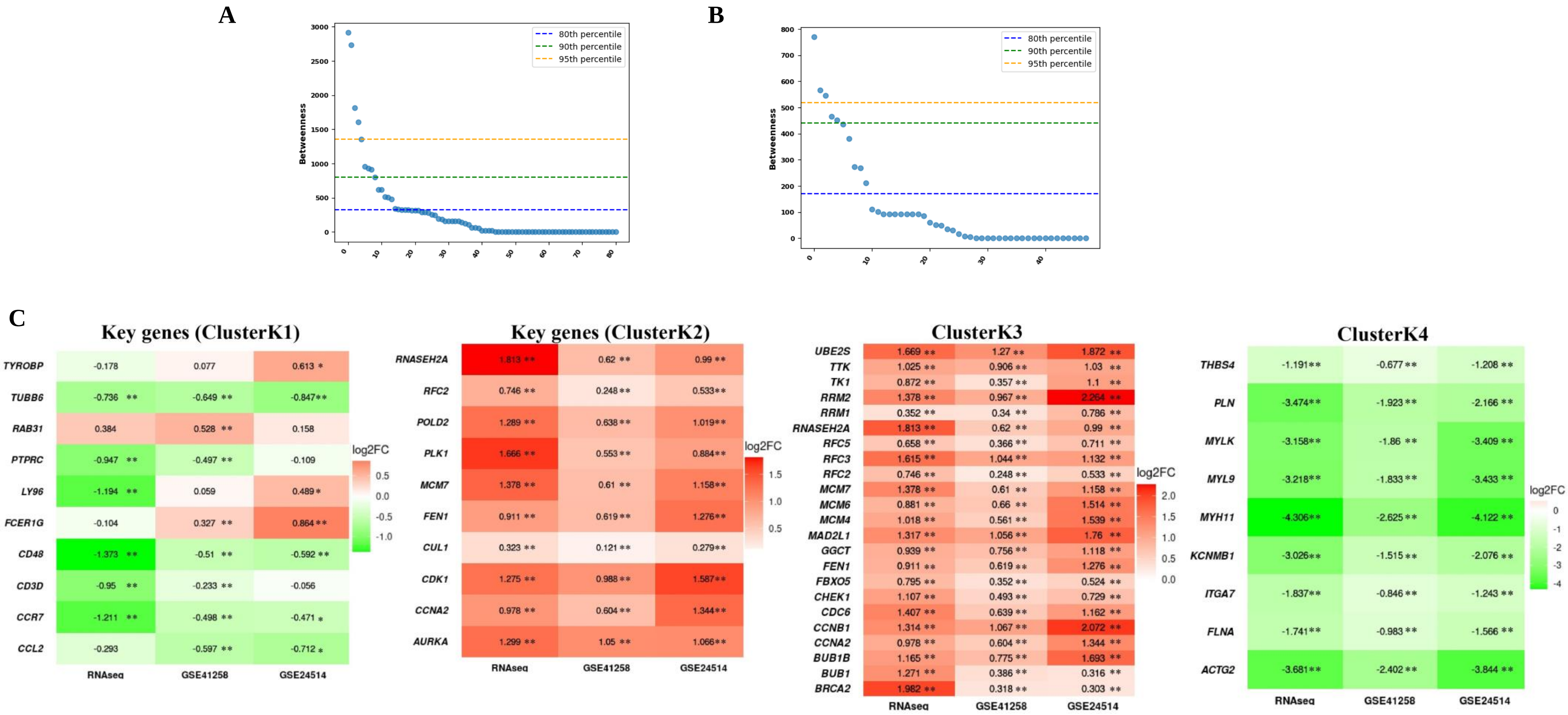


**Figure S3.** The frequency distribution of betweenness centrality for the PathGeNet **(A)** LCC 1 and **(B)** LCC 2 in CRC state. **(C)** The Heatmap representing the change in the expression (log2FC) of genes from normal to CRC state in the analysed independent datasets. The * represents the P-Value < 0.01 and ** represents the P-value <0.001.

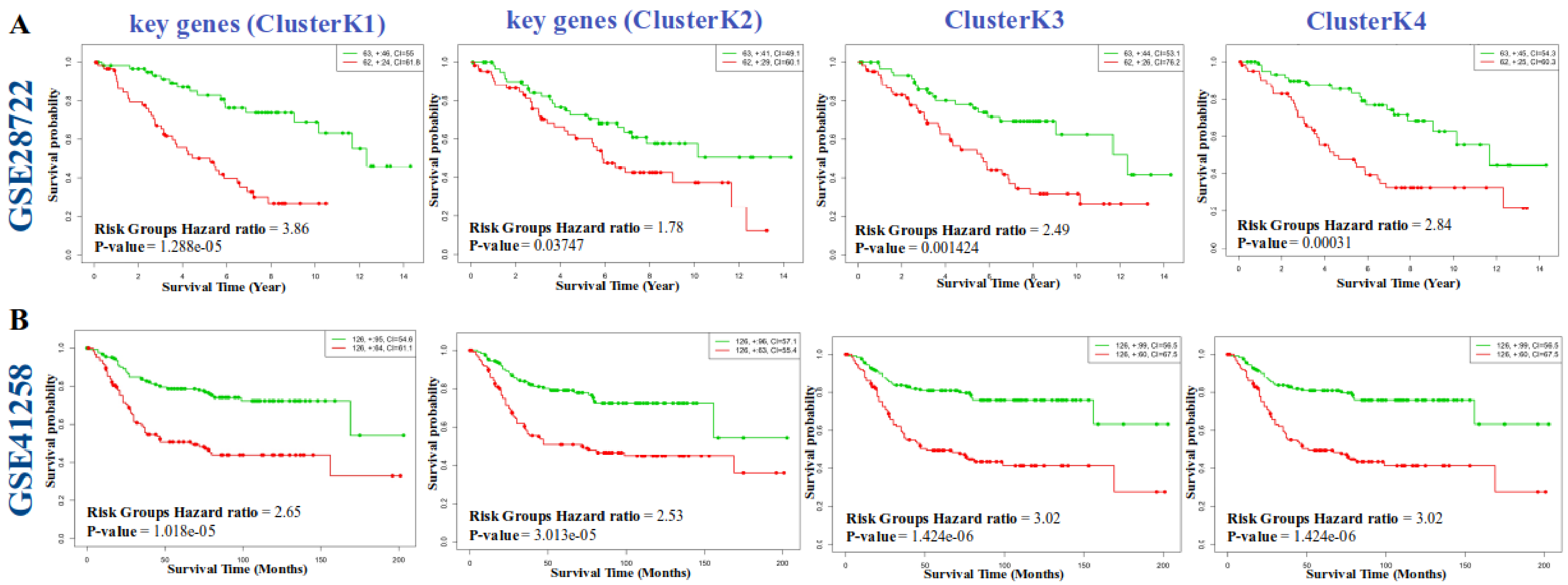


**Figure S4.** Survival analysis of the identified clusters in the independent gene expression datasets **(A)** GSE28722 and **(B)** GSE41258 datasets. The Red line representing the CRC patients with high-risk group and green line is representing the patients with low-risk group.

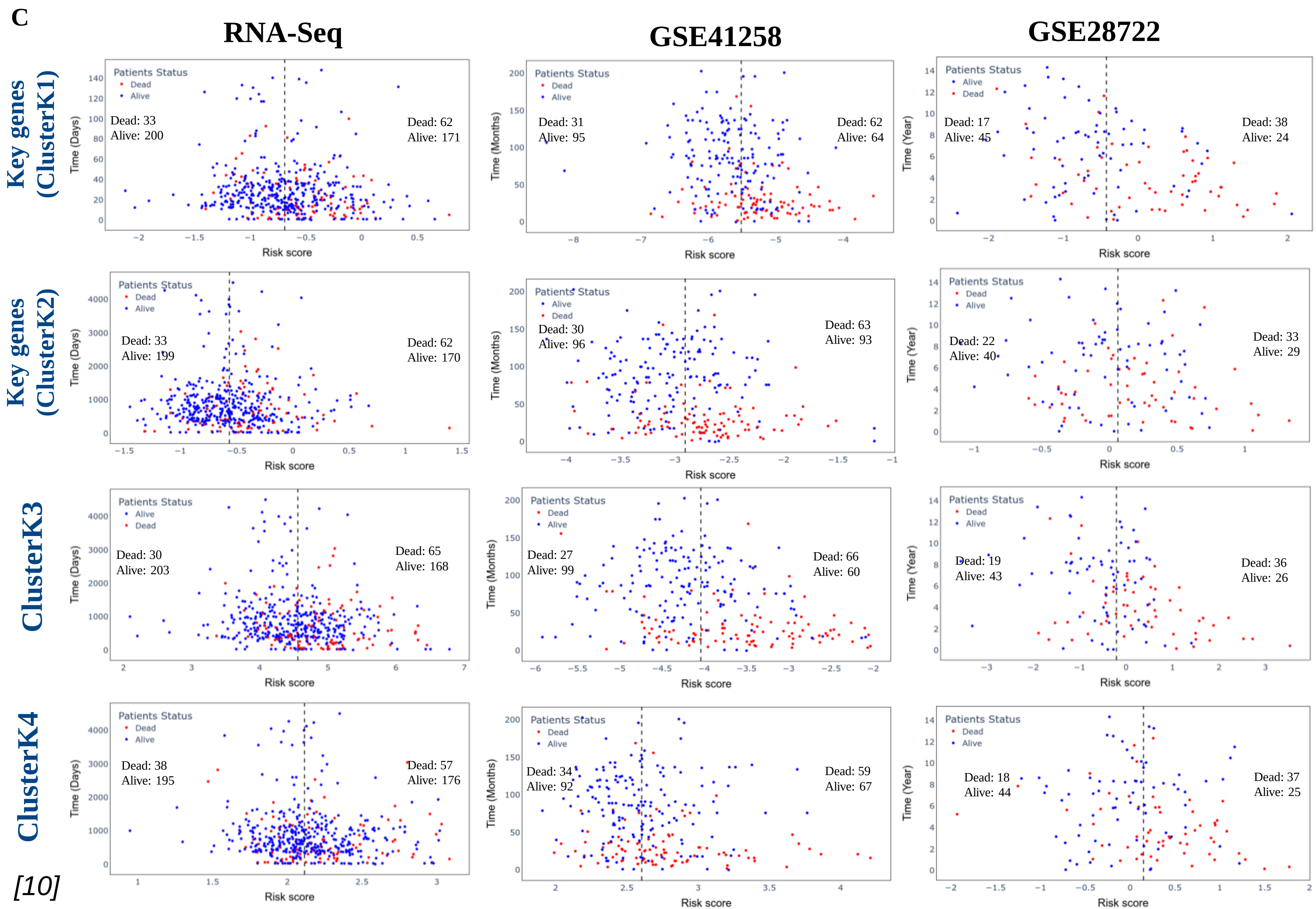


**Figure S4. (C)** Scatter plot representing the survival status of the CRC patients based on the risk score.

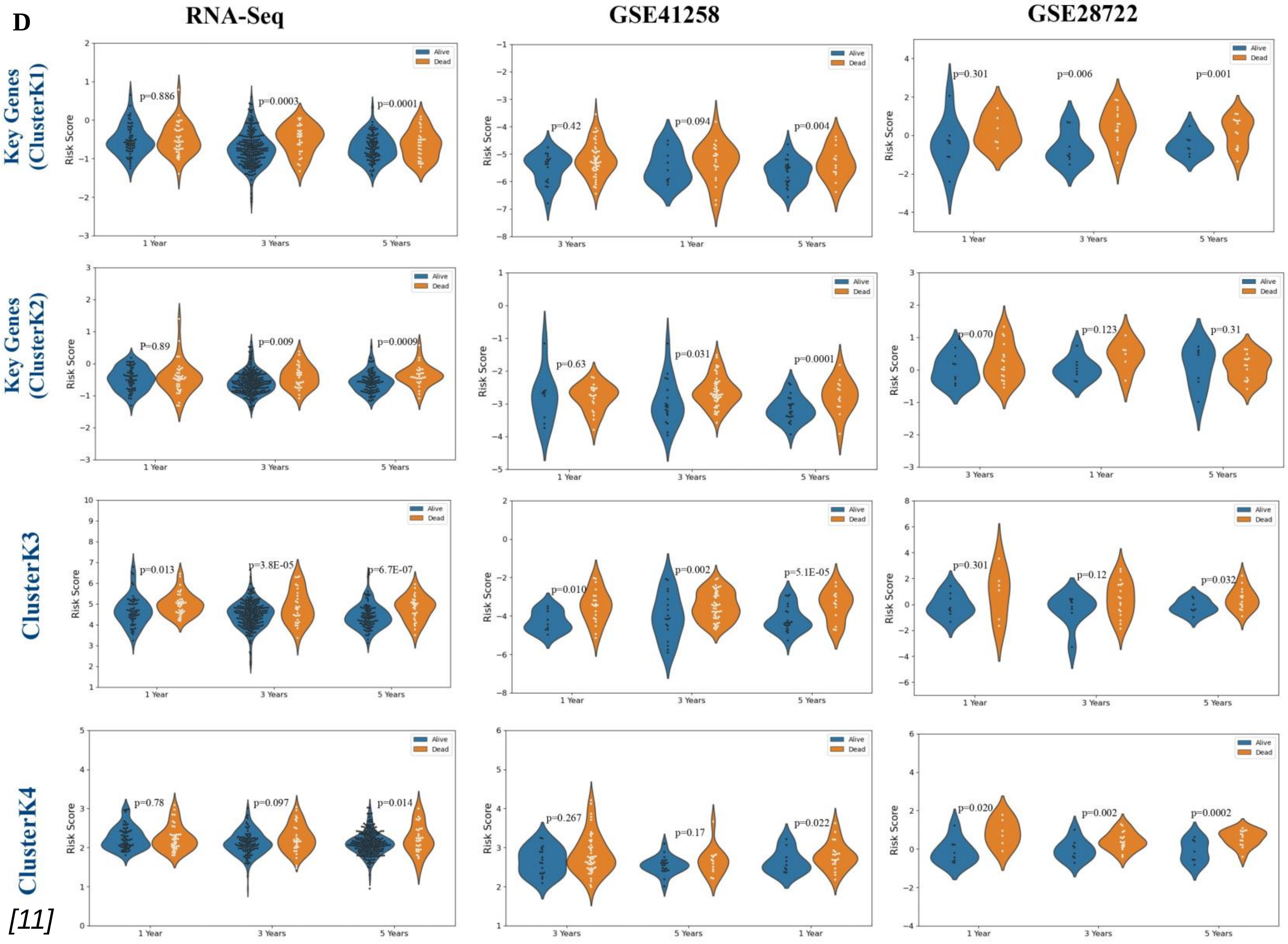


**Figure S4. (D)** Violin plot showing the distribution of risk scores between clinical status (Dead and Alive) for CRC patients at 1, 3, and 5 years.

**A**

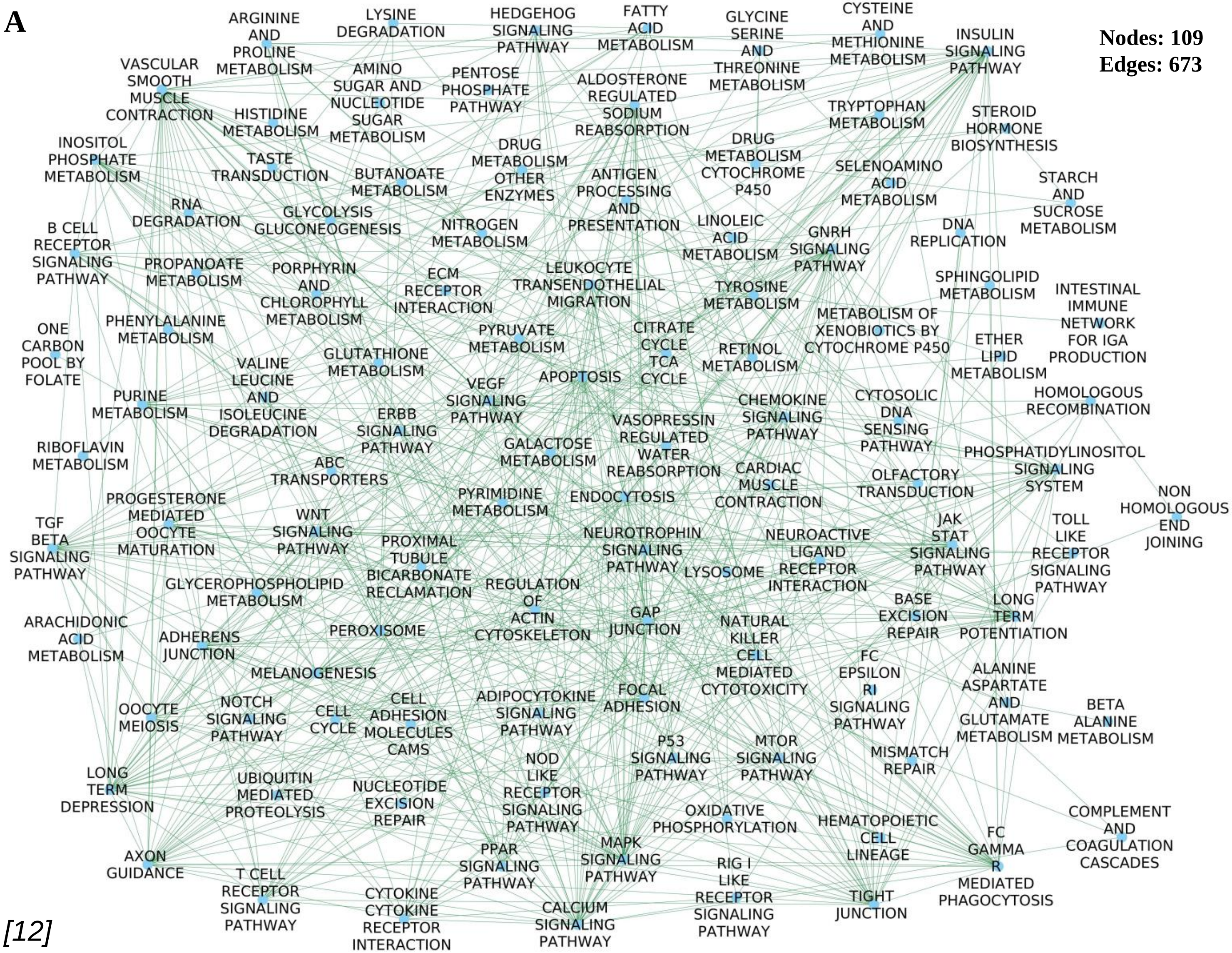


**Figure S5. (A)** Pathway cross-talk observed only in the normal state as compared to the CRC state.

**B**

**Nodes: 744**
**Edges: 1964**

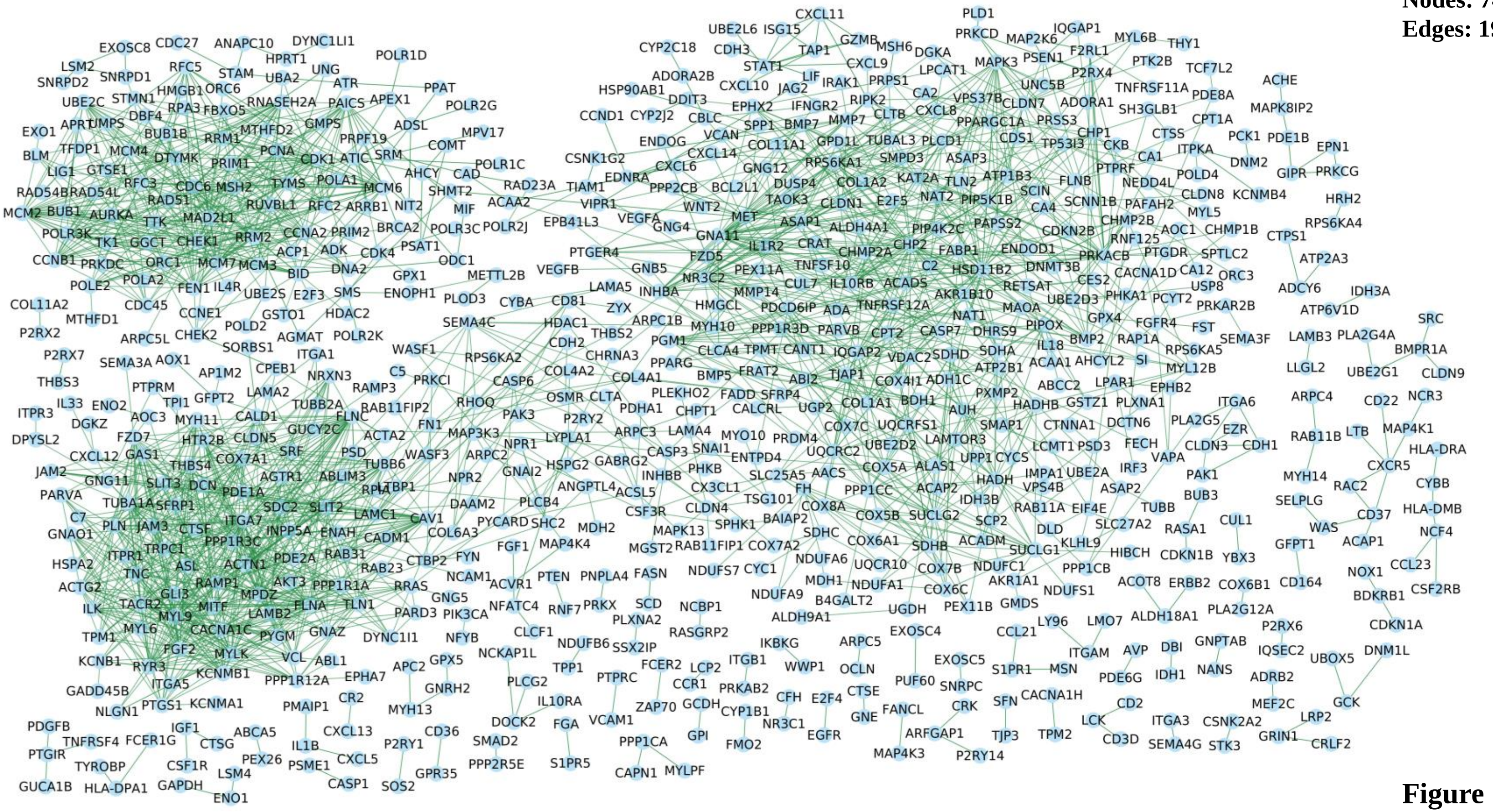

**Figure S5.** **(B)** The PathGeNet observed only in the normal state as compared to the CRC state.

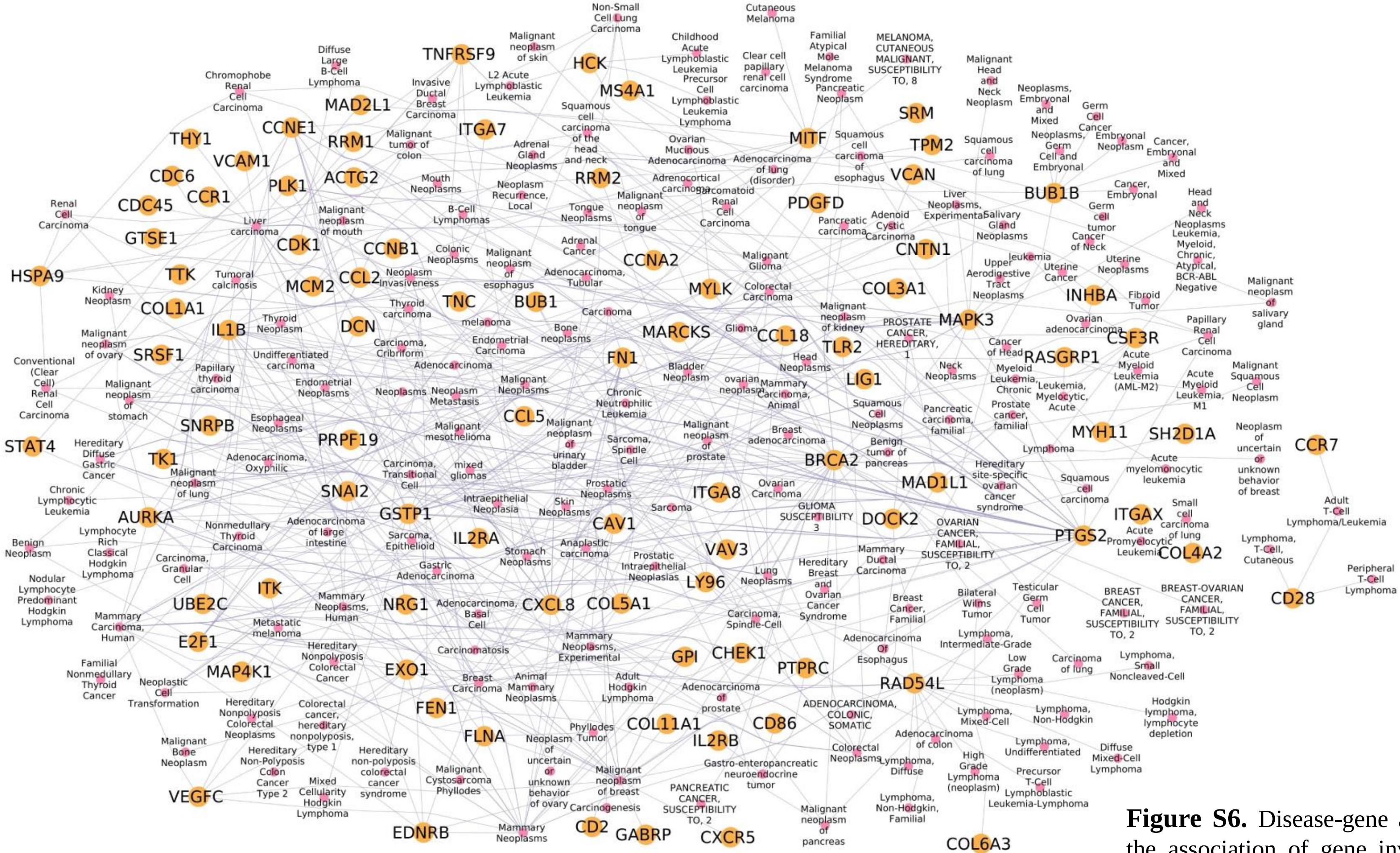


**Figure S6.** Disease-gene association network representing the association of gene involved in pathway cross-talk in CRC state with different cancer based on the curated DisGeNET database. The orange colour node representd gene involved in the Pathway cross-talk in the CRC state, pink colour nodes represents the different types of cancer.

# Section 2: Reactome pathway cross-talk analyses

Please refer '**Figure 1**' in the main text for the workflow

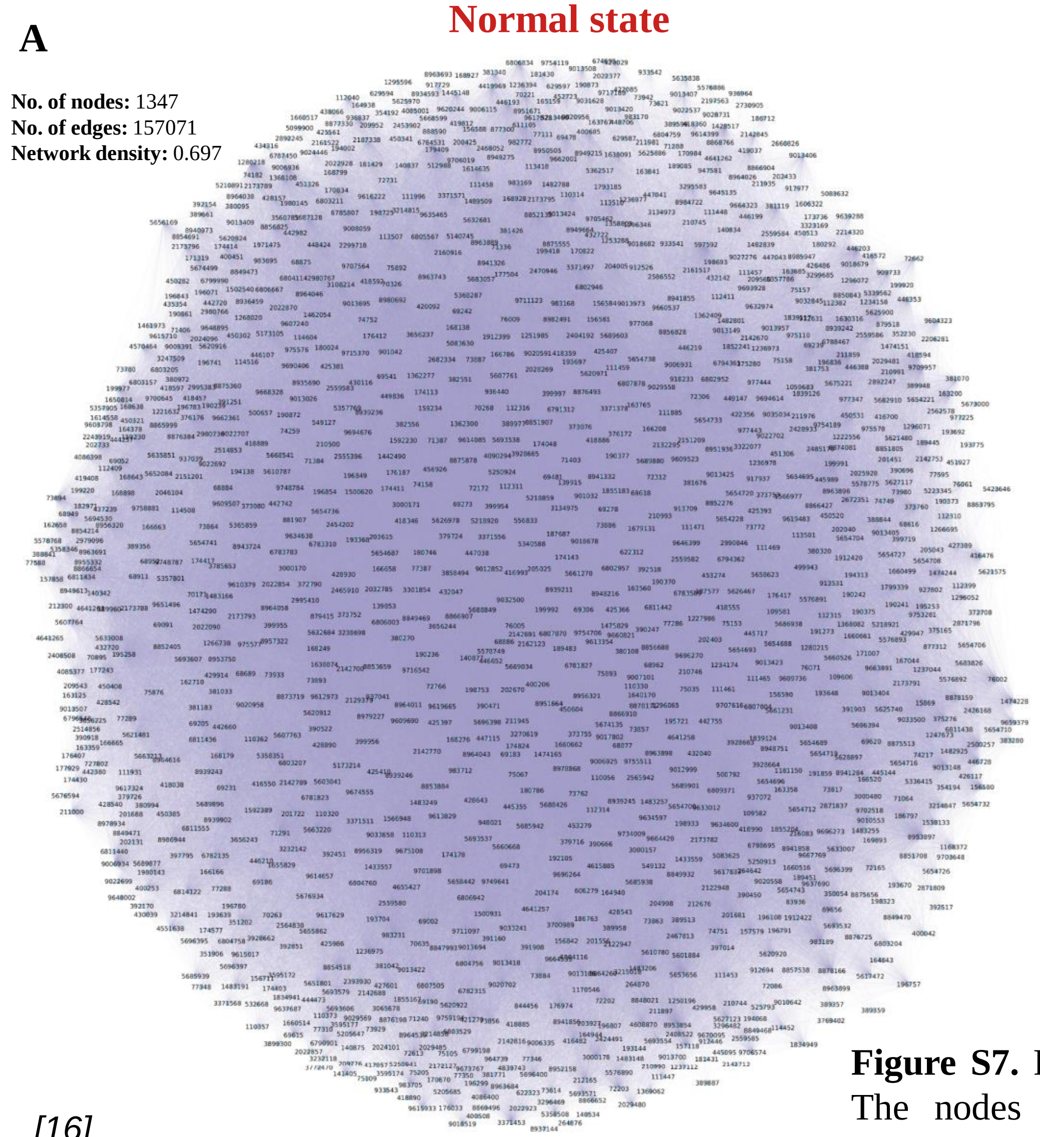


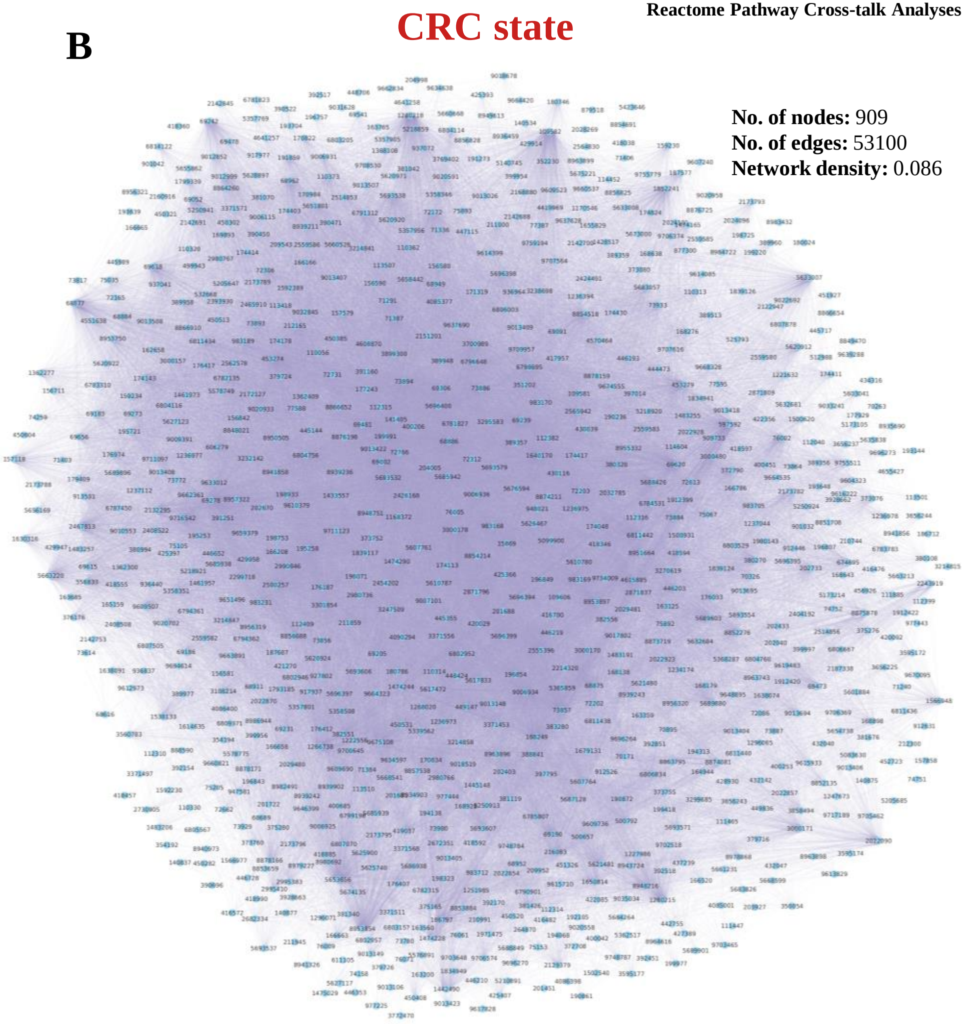


**Figure S7.** Pathway cross-talk network in **(A)** normal state and **(B)** CRC state. The nodes are representing the Reactome Pathway ID; example 1640170 represent the Reactome ID R-HSA-1640170 (cell cycle pathway).

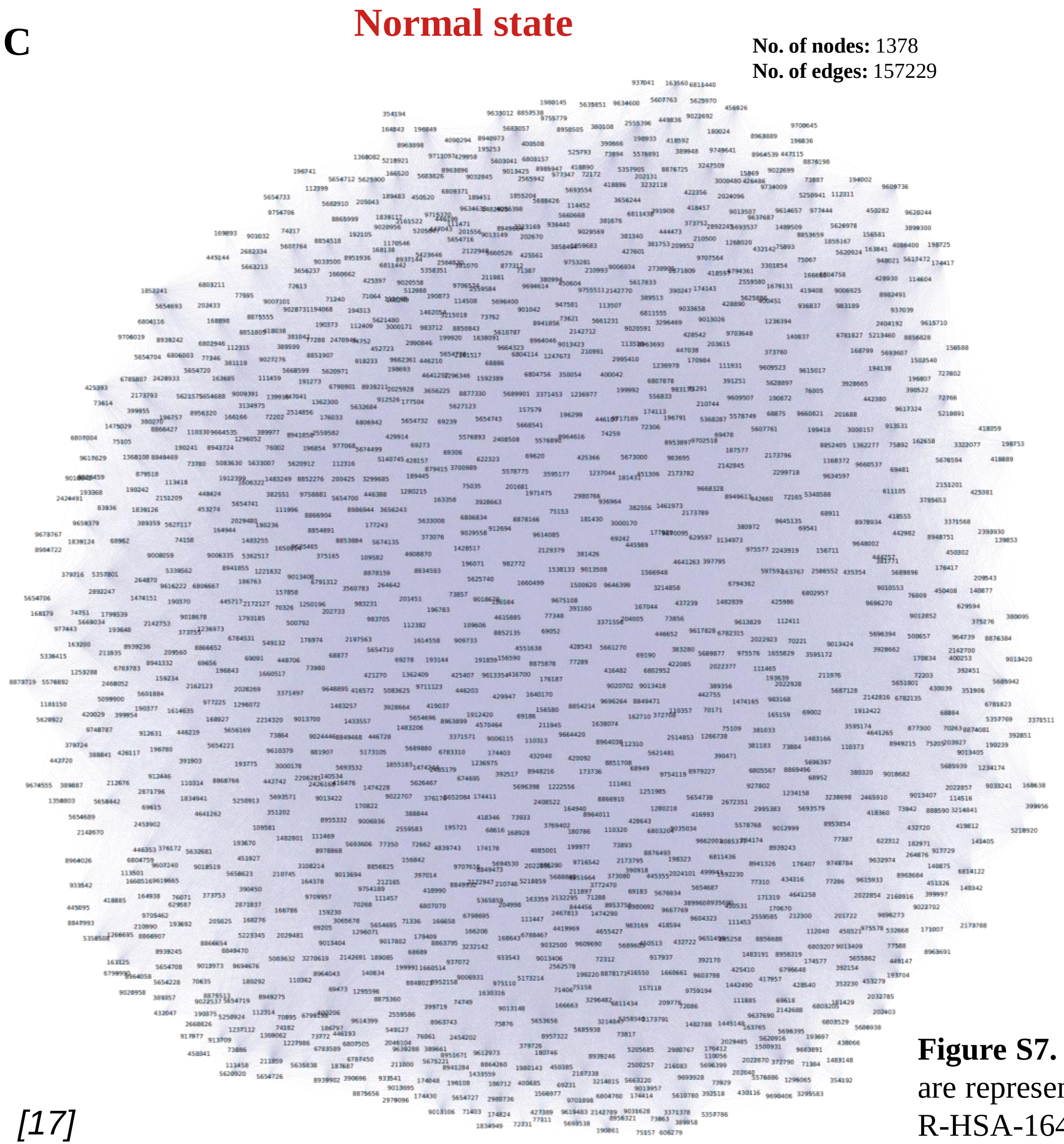


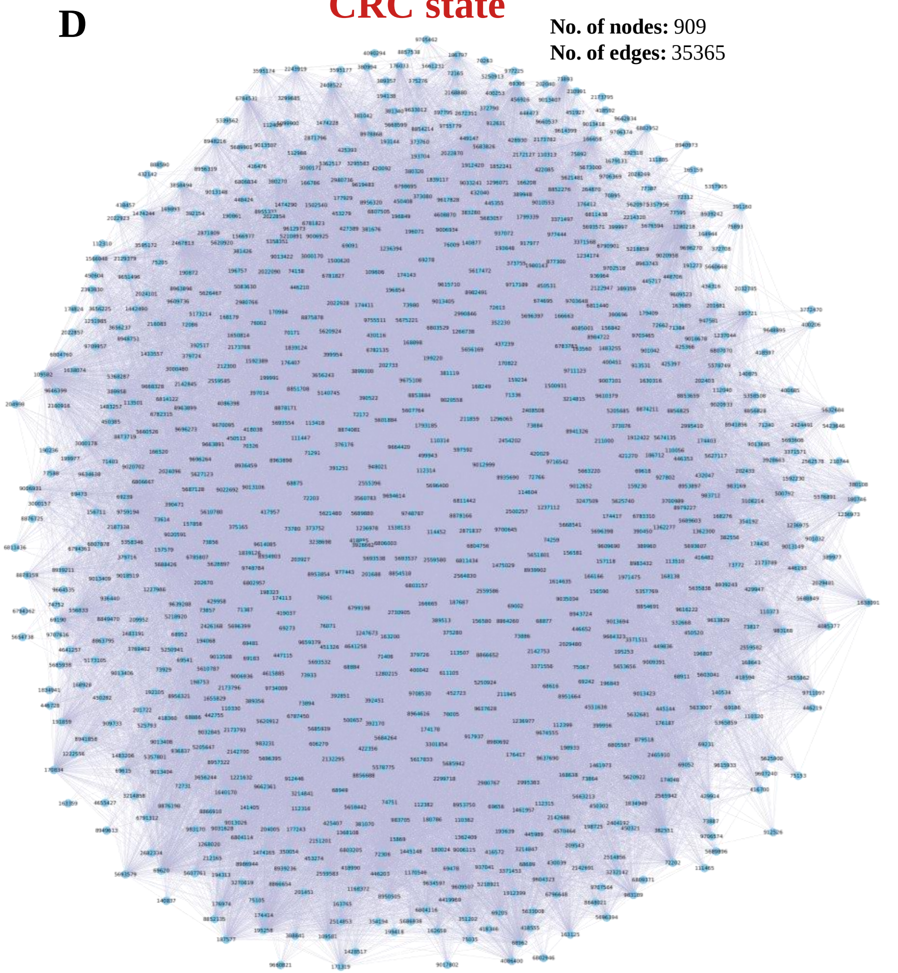


**Figure S7.** Unique pathway cross-talk in **(C)** Normal state and **(D)** CRC state. The nodes are representing the Reactome Pathway ID; example 1640170 represent the Reactome ID R-HSA-1640170 (cell cycle pathway).

**E**

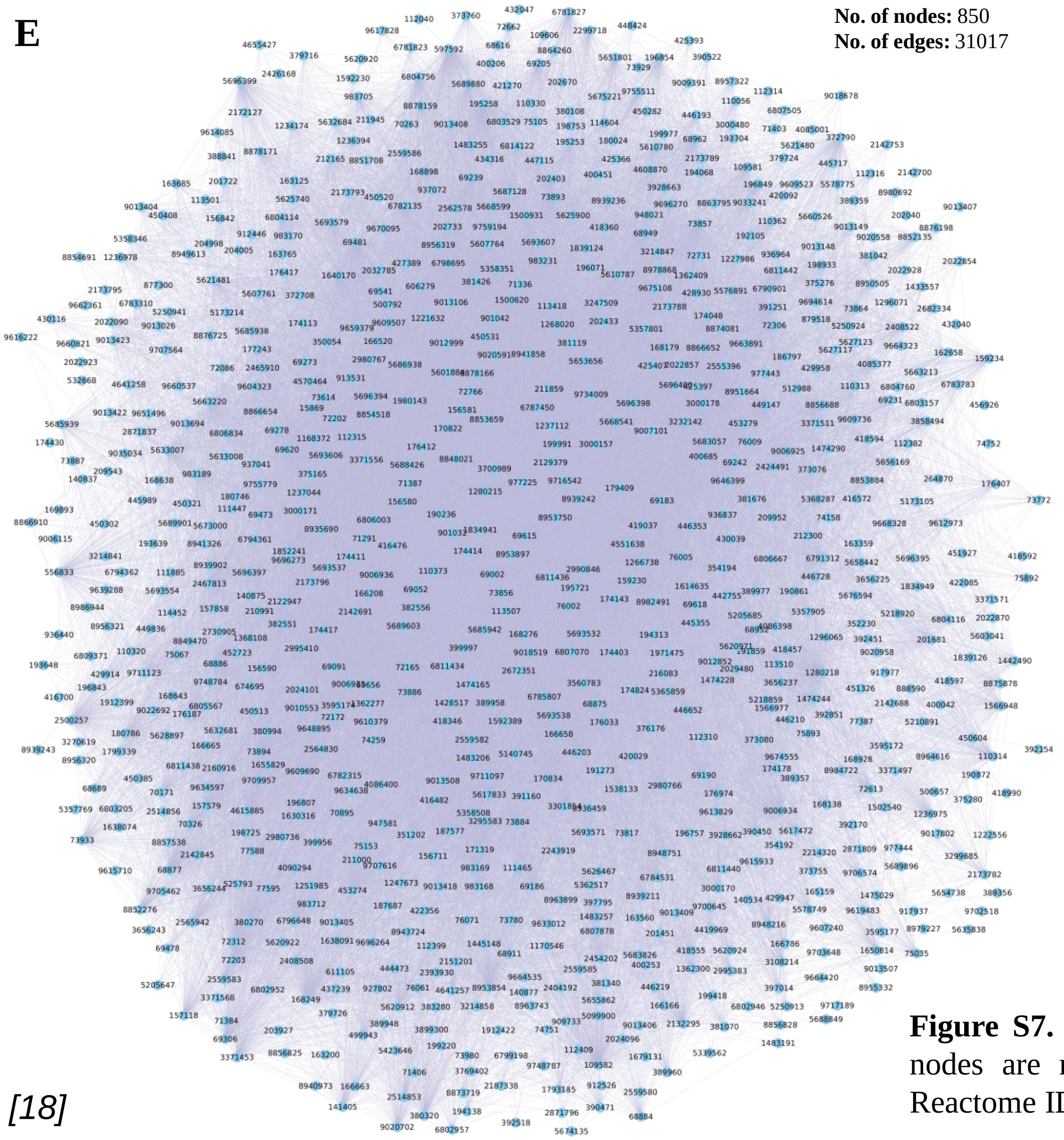


**Figure S7. (E)** Pathway cross-talk observed in both the normal and CRC state. The nodes are representing the Reatome Pathway ID; example 1640170 represent the Reactome ID R-HSA-1640170 (cell cycle pathway).

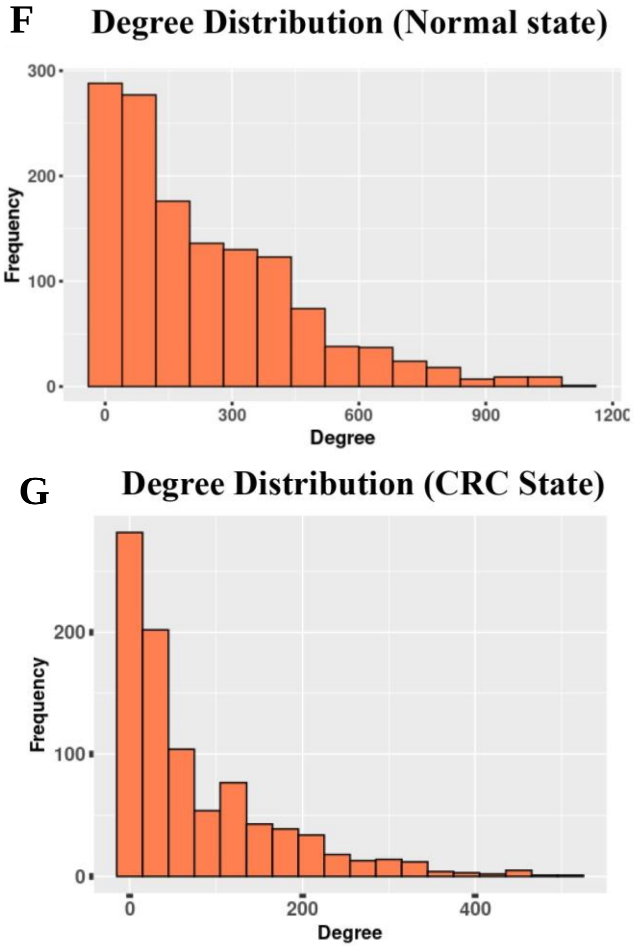


Top five pathways based on the pathway-pathway connectivity in normal state

| Rank | Pathway Name | Reactome ID | Degree |
|---|---|---|---|
| 1. | Post Translational Protein Modification | R-HSA-597592 | 1141 |
| 2. | Neutrophil Degranulation | R-HSA-6798695 | 1066 |
| 3. | Signaling by rho GTPases miro GTPases and rhobtb3 | R-HSA-9716542 | 1061 |
| 4. | Cellular Responses To Stimuli | R-HSA-8953897 | 1050 |
| 5. | Innate immune system | R-HSA-168249 | 1050 |

Top five pathways based on the pathway-pathway connectivity in CRC state

| Rank | Pathway Name | Reactome ID | Degree |
|---|---|---|---|
| 1. | Post Translational Protein Modification | R-HSA-597592 | 502 |
| 2. | Adaptive immune system | R-HSA-1280218 | 479 |
| 3. | Hemostasis | R-HSA-109582 | 465 |
| 4. | Cytokine Signaling In Immune System | R-HSA-1280215 | 454 |
| 5. | Innate immune system | R-HSA-168249 | 448 |

**Figure S7.** Reactome Pathway analysis: Histogram representing the degree distribution of the pathways and top five pathways based on the degree in the **(F)** normal and **(G)** CRC state.

**A**

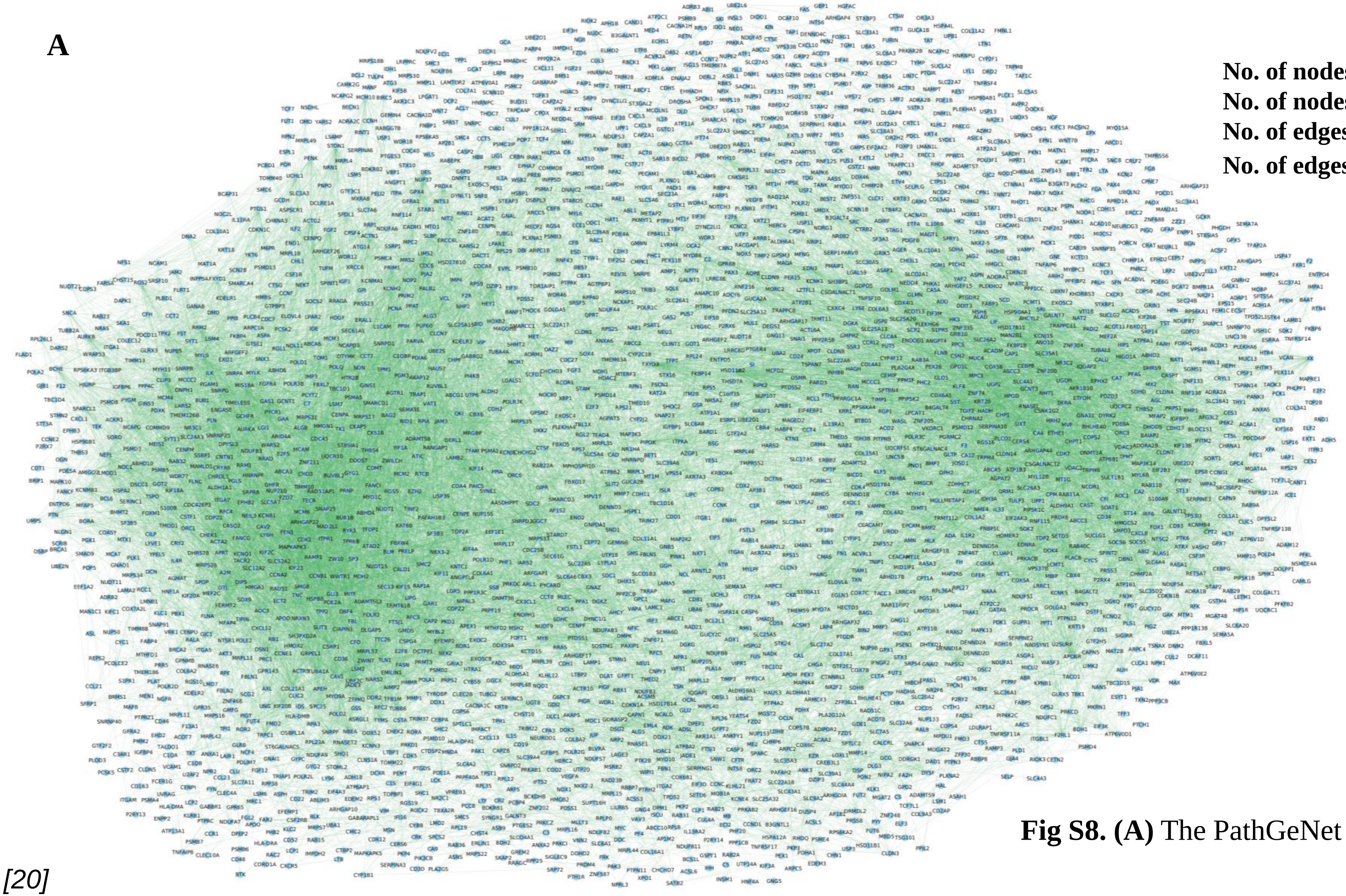

**No. of nodes:** 3041
**No. of nodes in LCC :** 2769
**No. of edges:** 19959
**No. of edges in LCC:** 19806

**Fig S8. (A)** The PathGeNet in Normal state.

**B**

**No of nodes:** 1211
**No of edges:** 2685

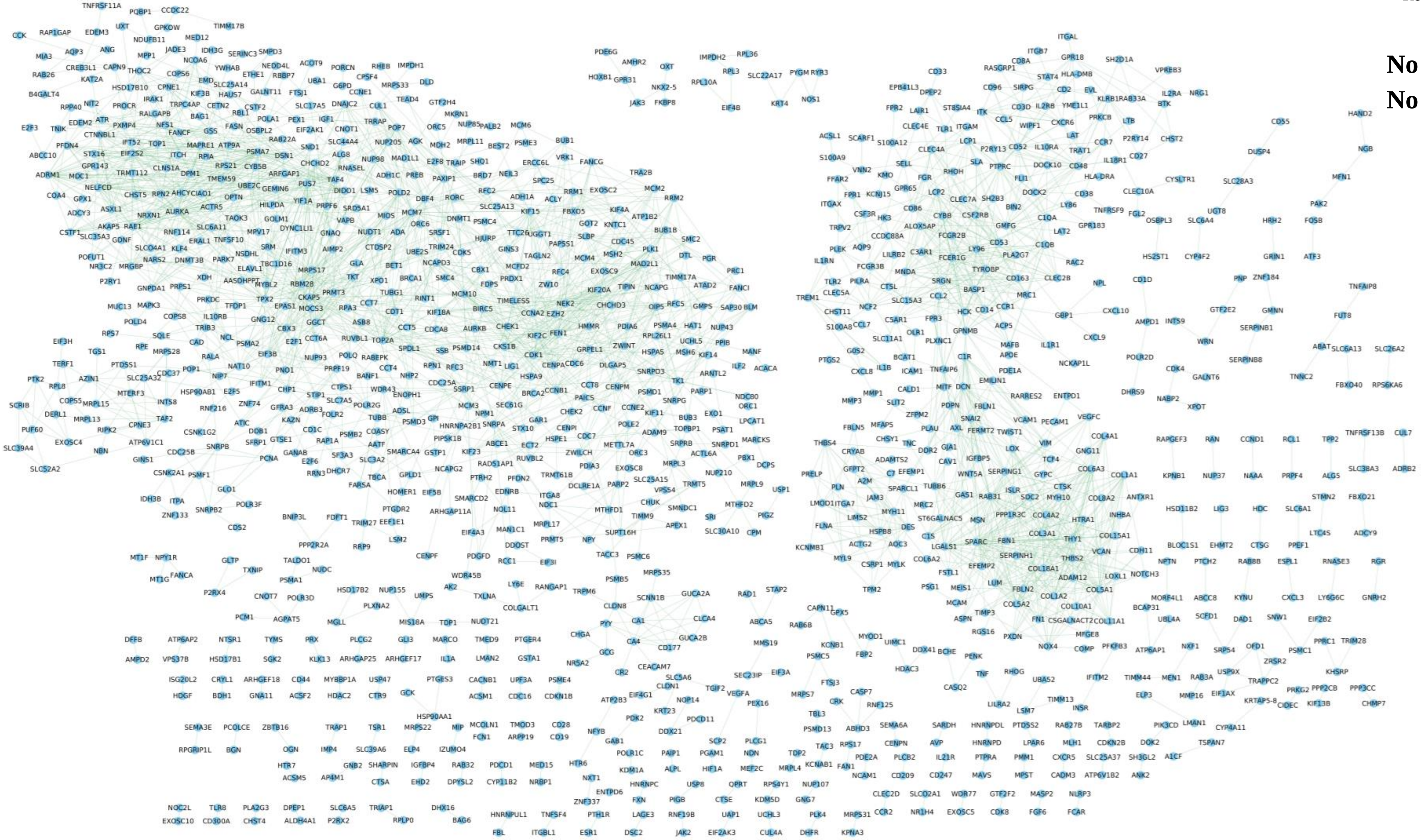

**Figure S8. (B)** The PathGeNet in CRC state.

# Comparative study of PathGeNet (pathway cross-talk interface gene co-expression network): KEGG vs. Reactome

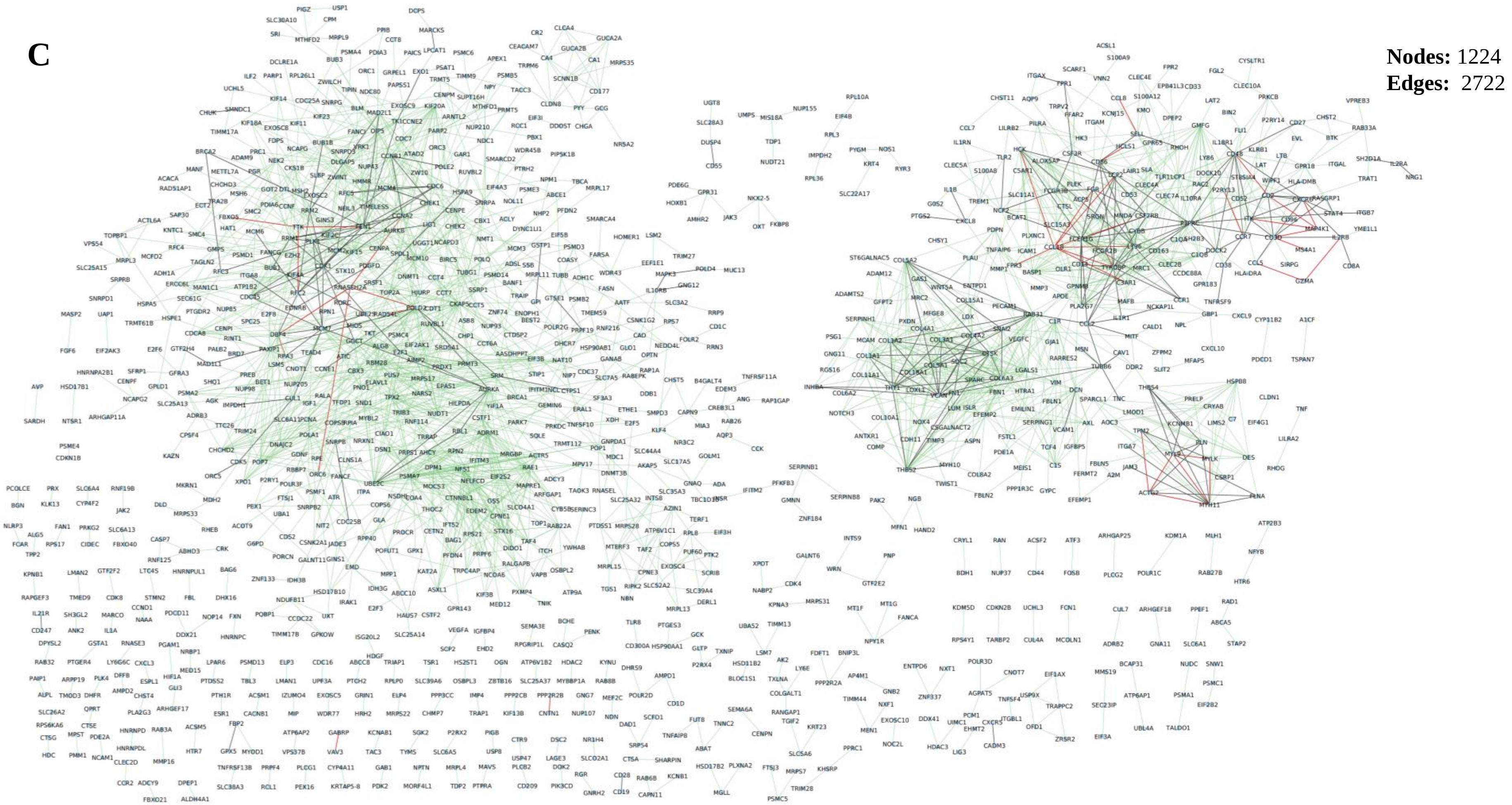


**Figure S8. (C)** Comparative study of PathGeNet of the KEGG and Reactome in CRC state. Red edges represent gene pairs unique to the KEGG pathway cross-talk, green edges to the Reactome pathway cross-talk, and gray edges shared between both KEGG and Reactome pathway cross-talks.

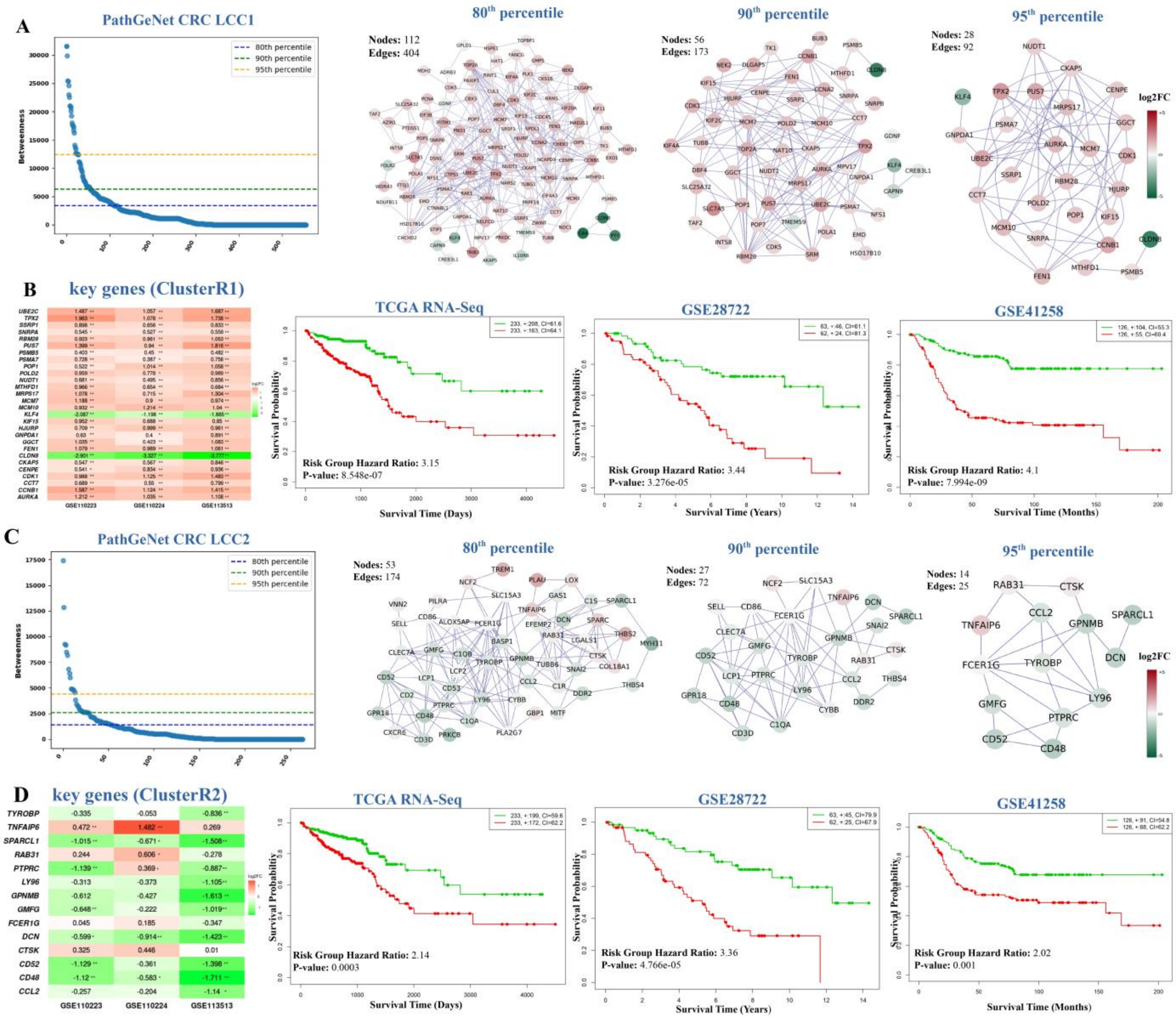


**Figure S9. (A & C)** Key genes identified from the PathGeNet analysis in CRC state and **(B & D)** heatmap representing the changes in gene expression of these key genes and their impact on the survival of CRC patients. In heatmap, * represents the P-value <0.05 and ** represents the P-value <0.01.

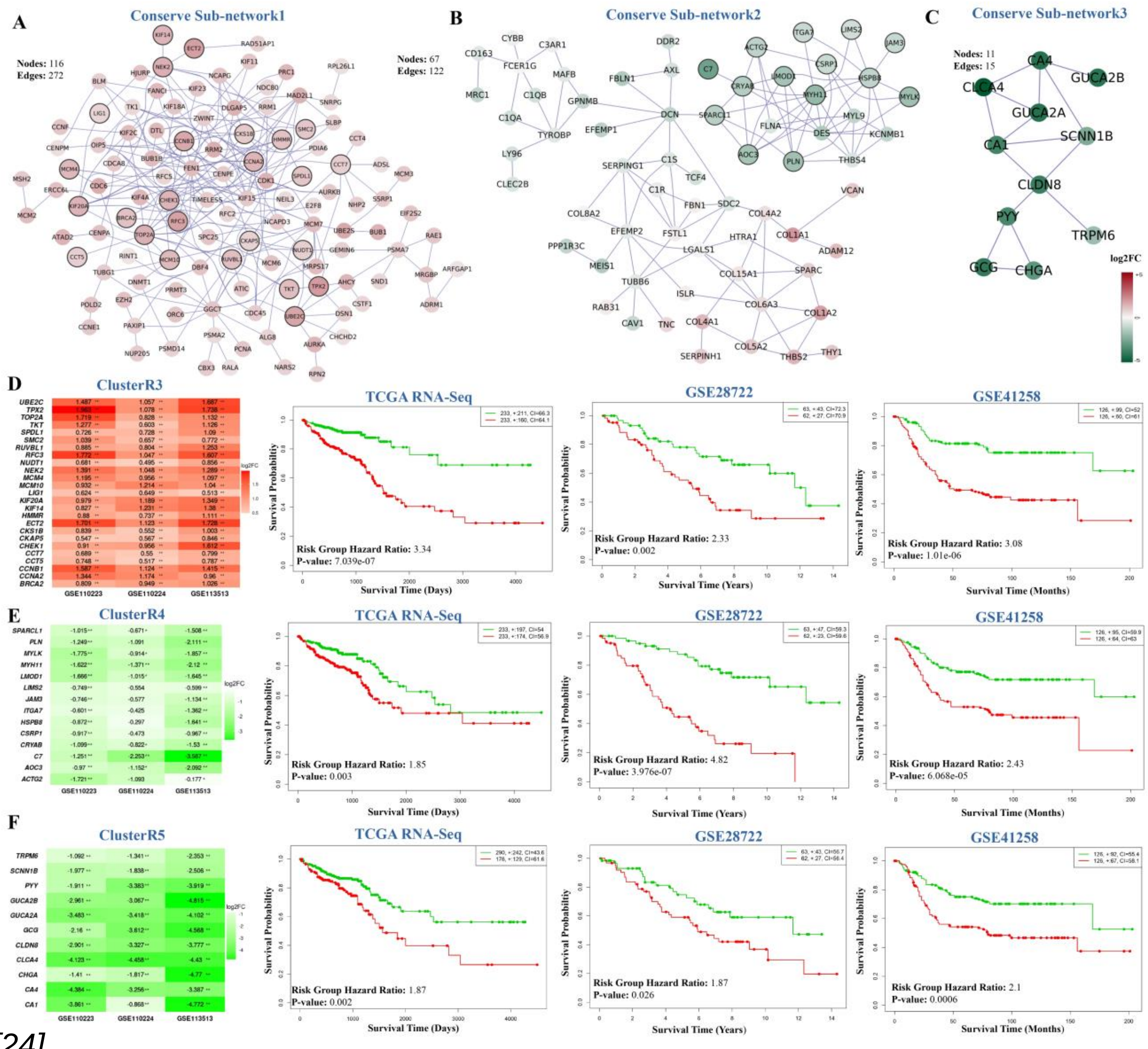


**Figure S10. (A-C)** The conserve sub-networks from the PathGeNet between the normal and CRC states. The nodes highlighted with a black boundary are part of the selected Clusters. **(D-E)** Representing the heatmap (log2FC) and survival analysis of the identified ClustersR3 and ClsuterR4. **(F)** heatmap and survival analysis of ClusterR5. In heatmap, * represents the P-value <0.05 and ** represents the P-value <0.01.

† SPDL1 from sub-network of ClusterR3 and two genes GUCA2A and PYY from ClusterR5 were missing in the GSE28722 datasets for survival analysis. SPDL1 in TCGA RNA-Seq datasets is also missing.

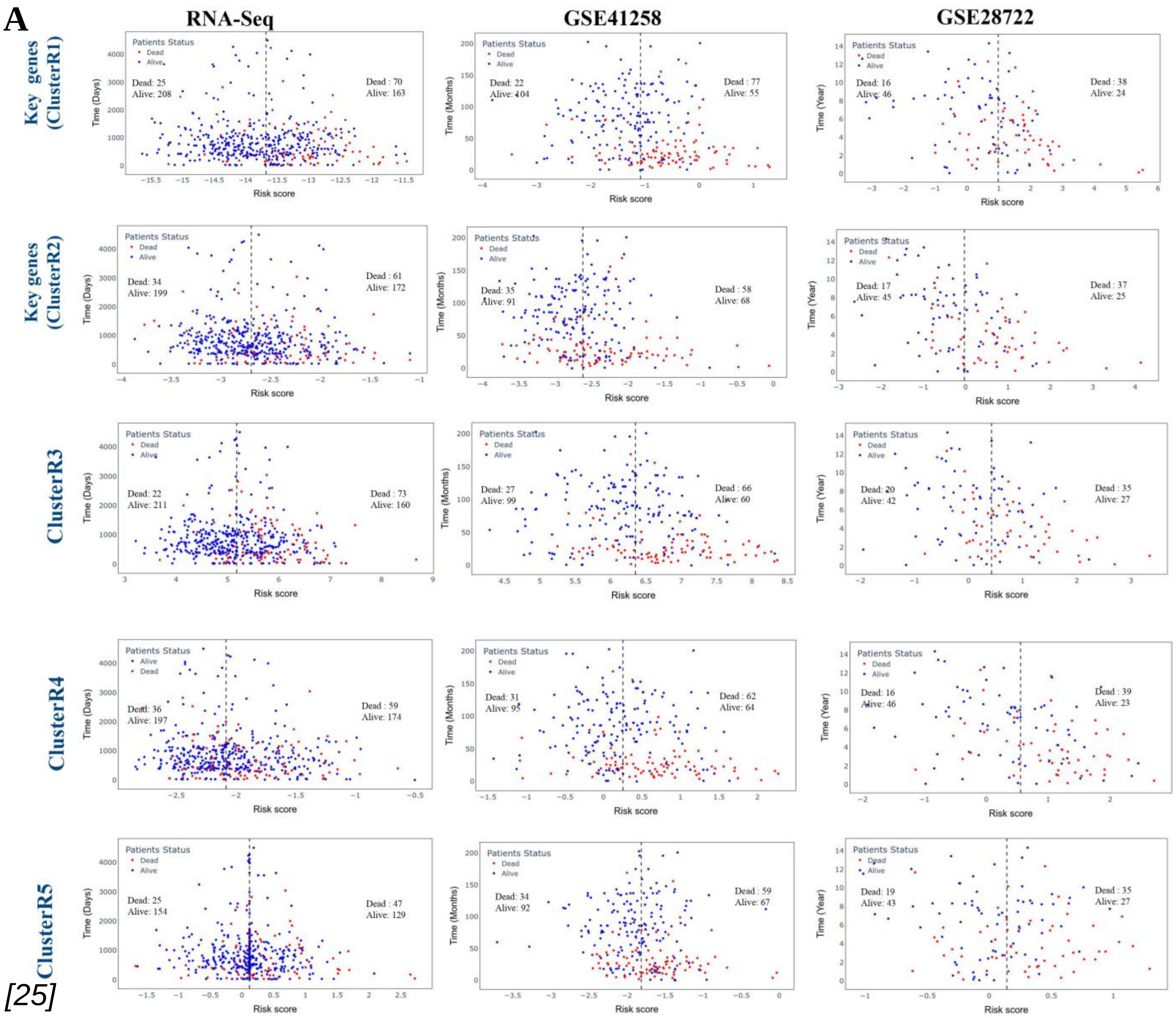


**Figure S11. (A)** Scatter plot representing the survival status of the CRC patients based on the risk score.

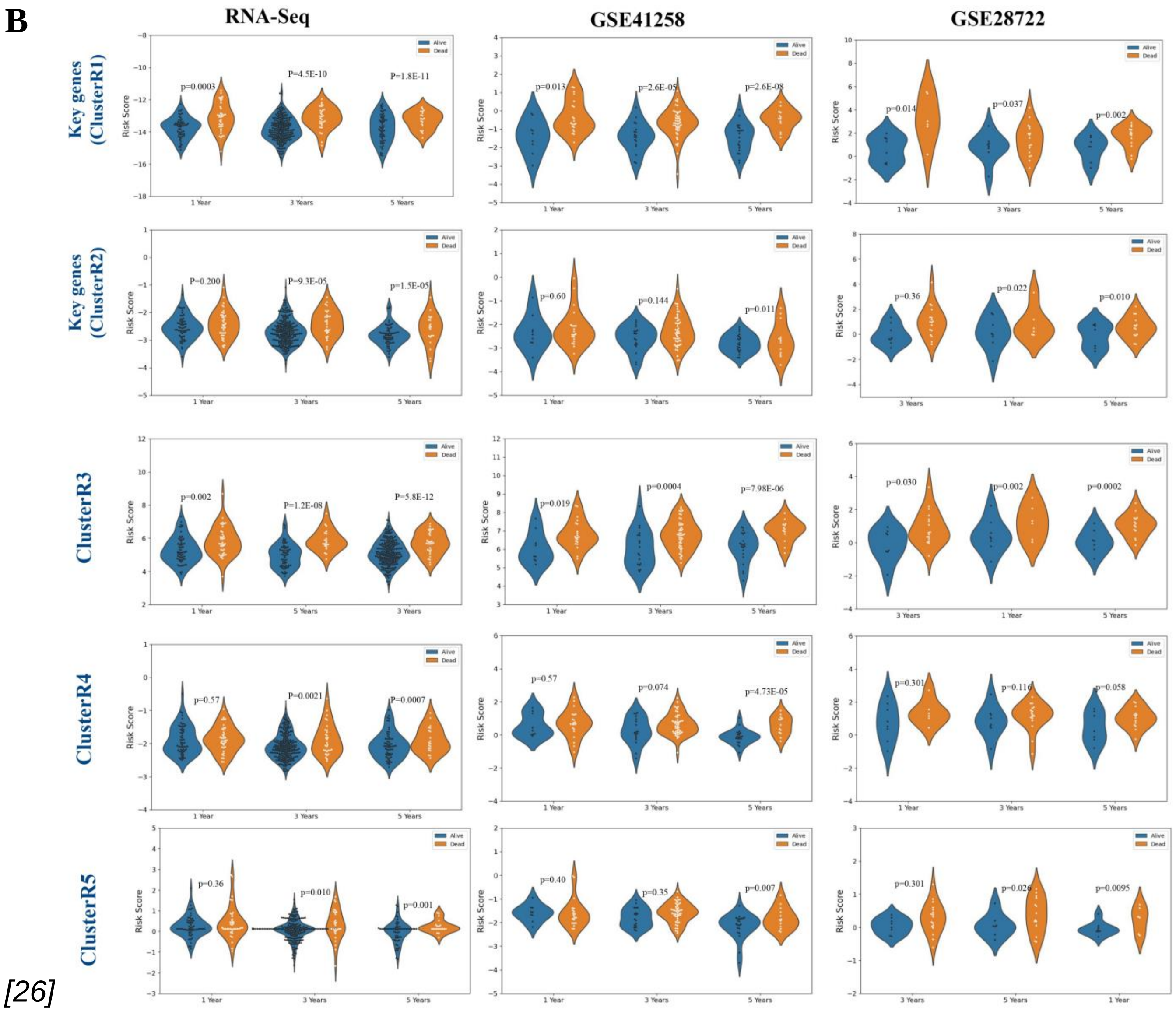


**Figure S11. (B)** Violin plot showing the distribution of risk scores between clinical status (Dead and Alive) for CRC patients at 1, 3, and 5 years.

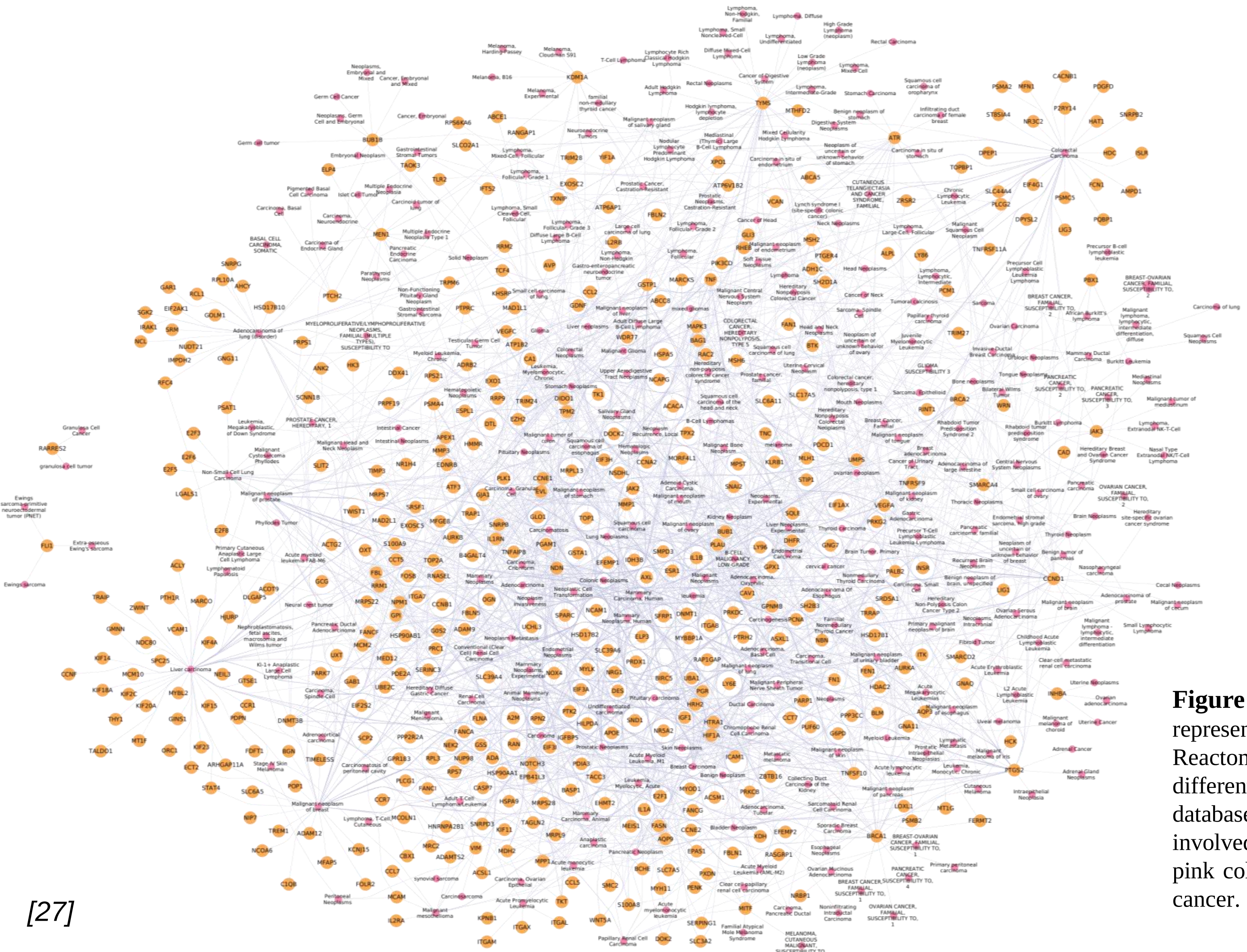

**Figure S12.** Disease-gene association network representing the association of gene involved in Reactome pathway cross-talk in CRC state with different cancer based on the curated DisGeNET database. The orange colour node representd gene involved in the Pathway cross-talk in the CRC state, pink colour nodes represents the different types of cancer.

# Section 3: Gene regulatory network analyses

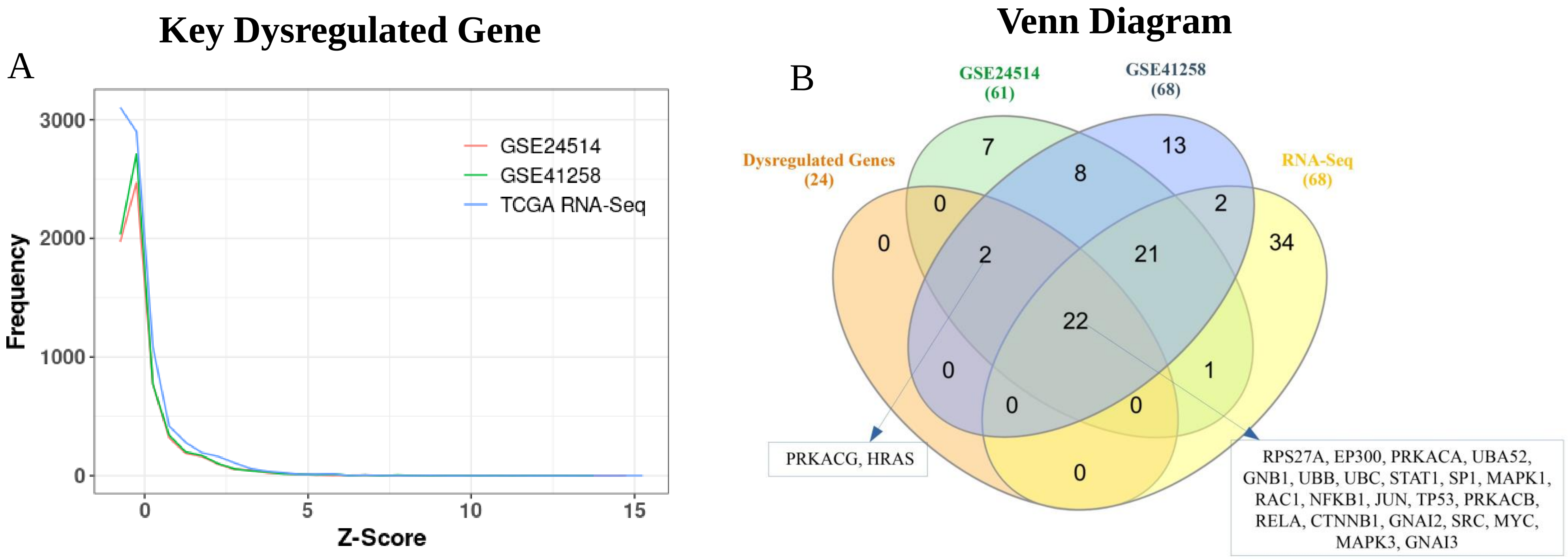


**Figure S13.** Validation of dysregulated genes: **(A)** Distribution of dysregulated scores (Z-scores) across three independent datasets. **(B)** Venn diagram illustrating the overlap of identified dysregulated genes in the independent datasets with the analyzed dataset

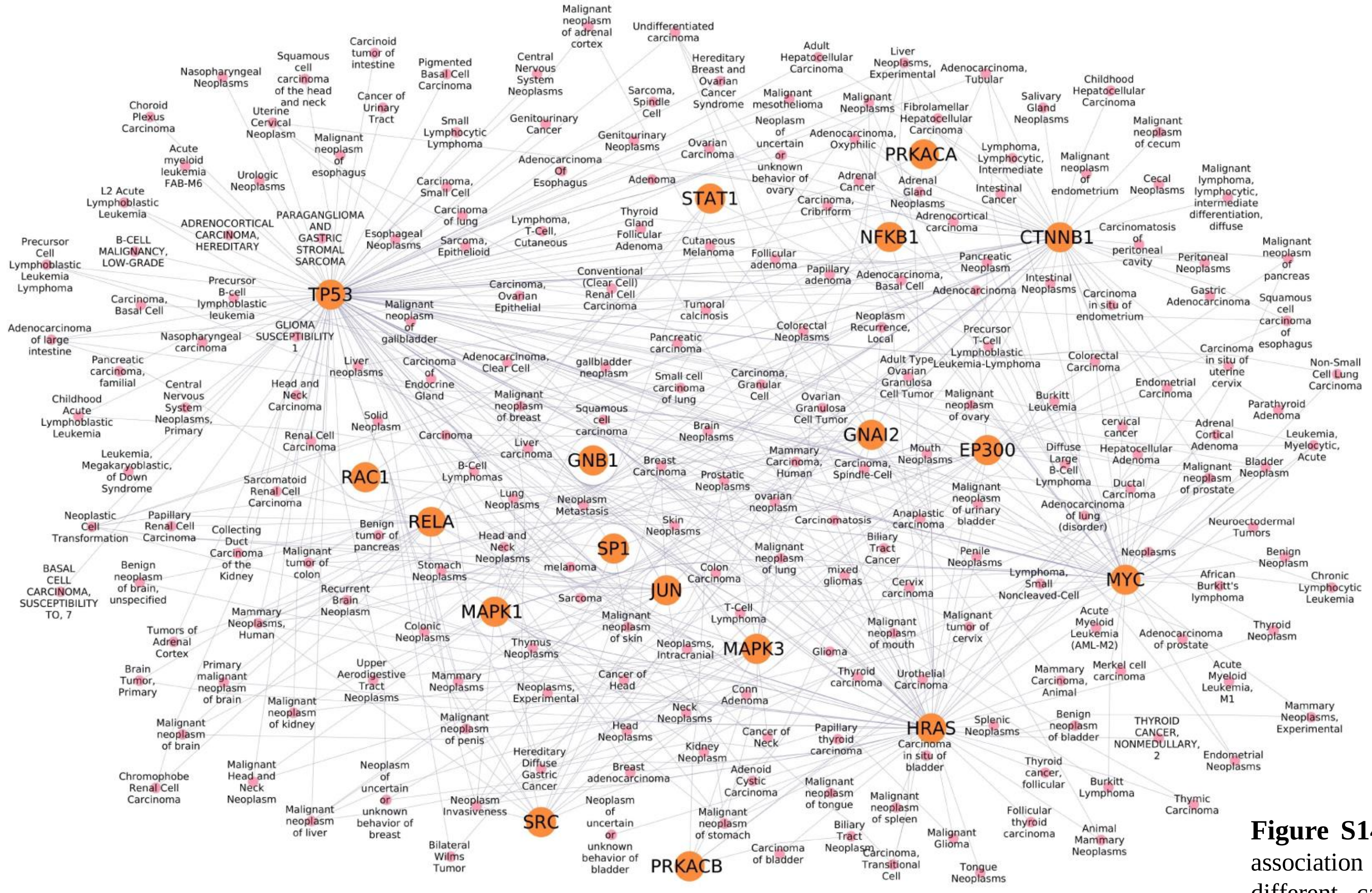


**Figure S14.** Disease-gene network representing the association identifed key dysregulated genes with different cancer based on the curated DisGeNET database. The orange colour node representd key dysregulated genes, pink colour nodes represents the different types of cancer .

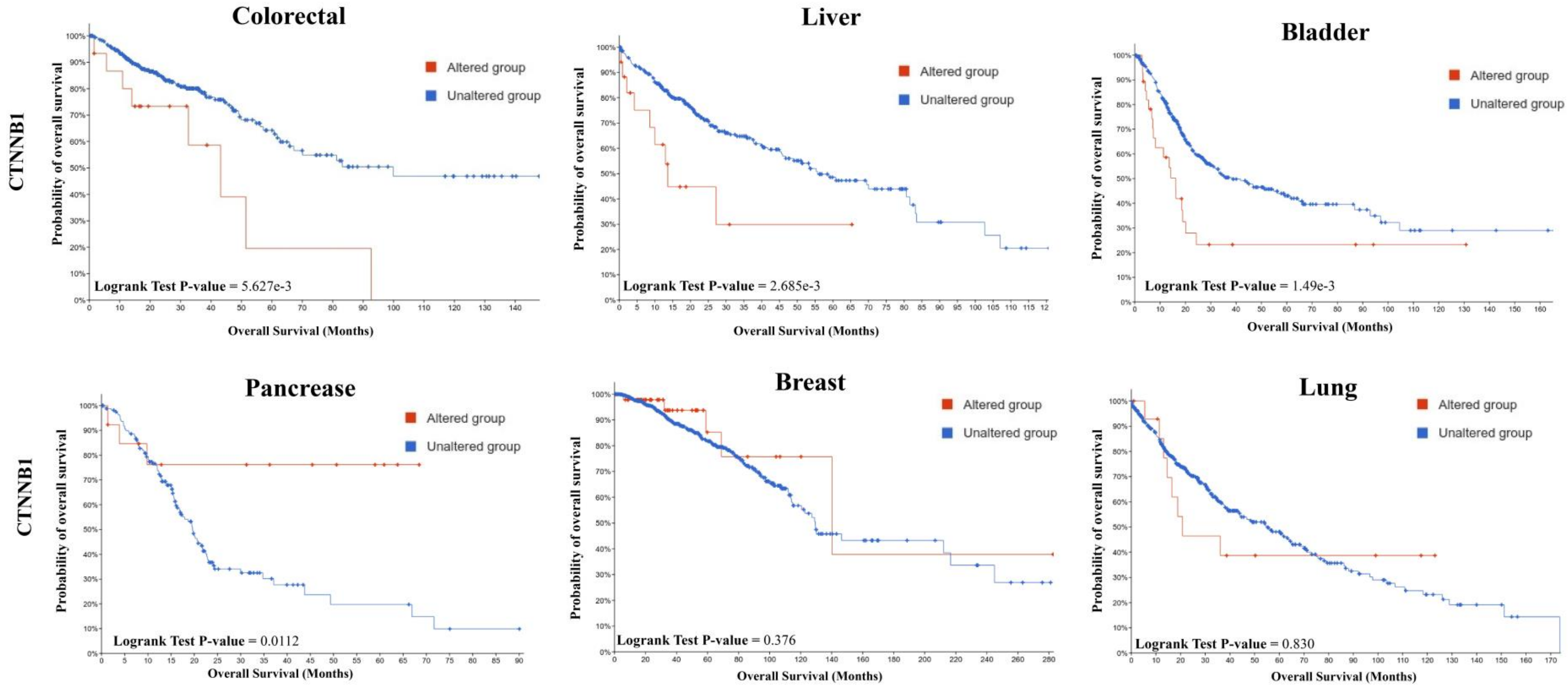


**Figure S15.** Survival analysis results representing the prognostic significance of the CTNNB1 gene in the CRC and other different tissues.

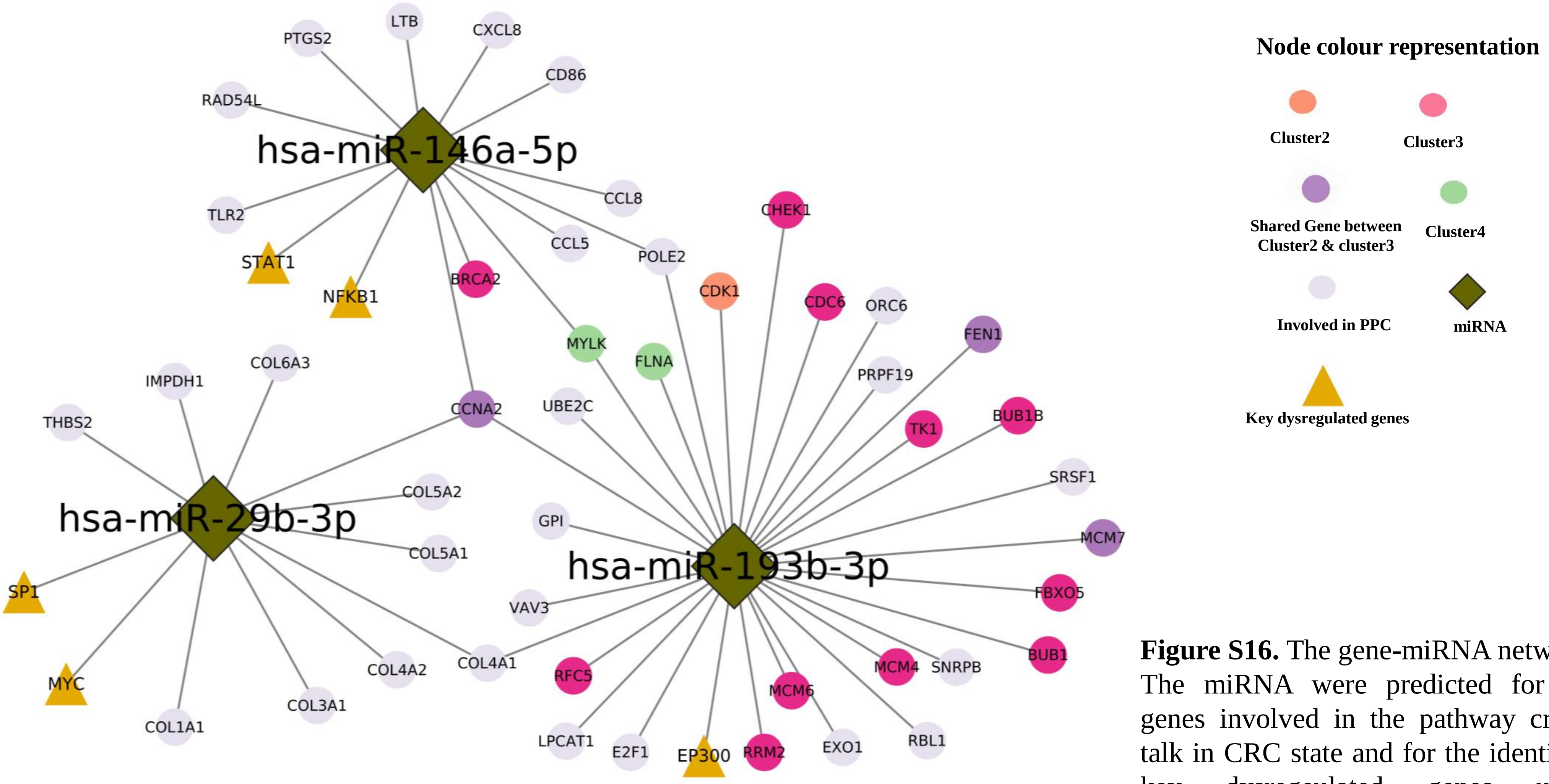


**Figure S16.** The gene-miRNA network. The miRNA were predicted for the genes involved in the pathway cross-talk in CRC state and for the identified key dysregeulated genes using g:Profiler (http://biit.cs.ut.ee/gprofiler/) web server.

# Section 4: Potential repurposed drug molecules

**Drug Name:** DL-PDMP
**PubChem Id:** 16219895

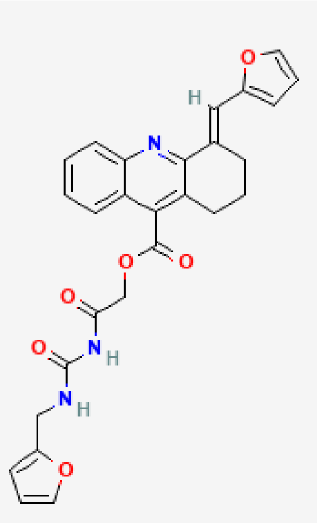

**Drug Name:** GDC-0980
**PubChem Id:** 25254071

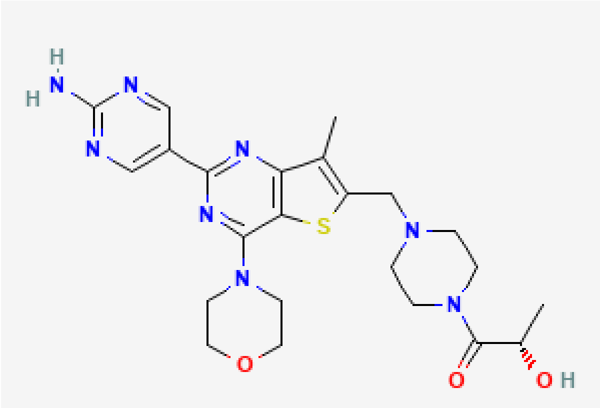

**Drug Name:** CAM-9-027-3
**PubChem Id:** 49849912

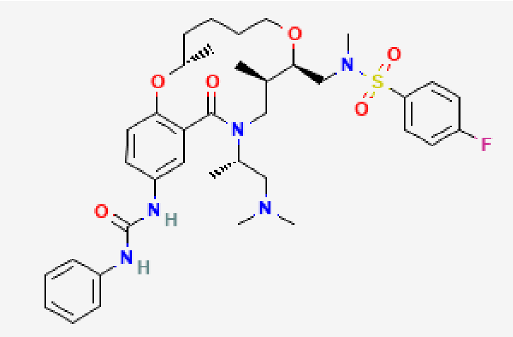

**Drug Name:** Foretinib
**PubChem Id:** 42642645

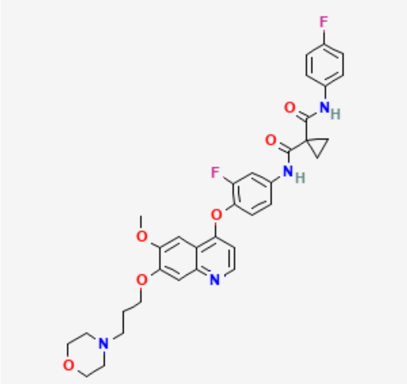

**Drug Name:** PF 750
**PubChem Id:** 25154868

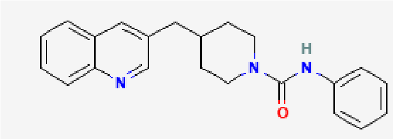

**Drug Name:** WH-4-025
**PubChem Id:** 73707529

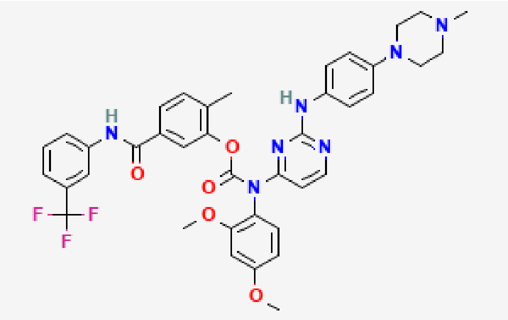

**Drug Name:** Palbociclib
**PubChem Id:** 5330286

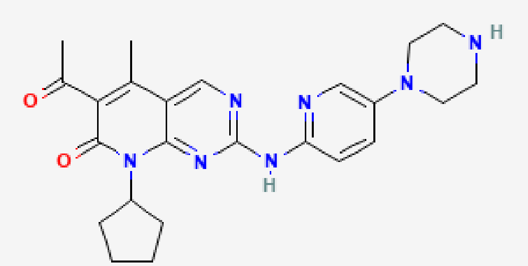

**Figure S17.** Structure of identified potentail drug candidates for the CRC.

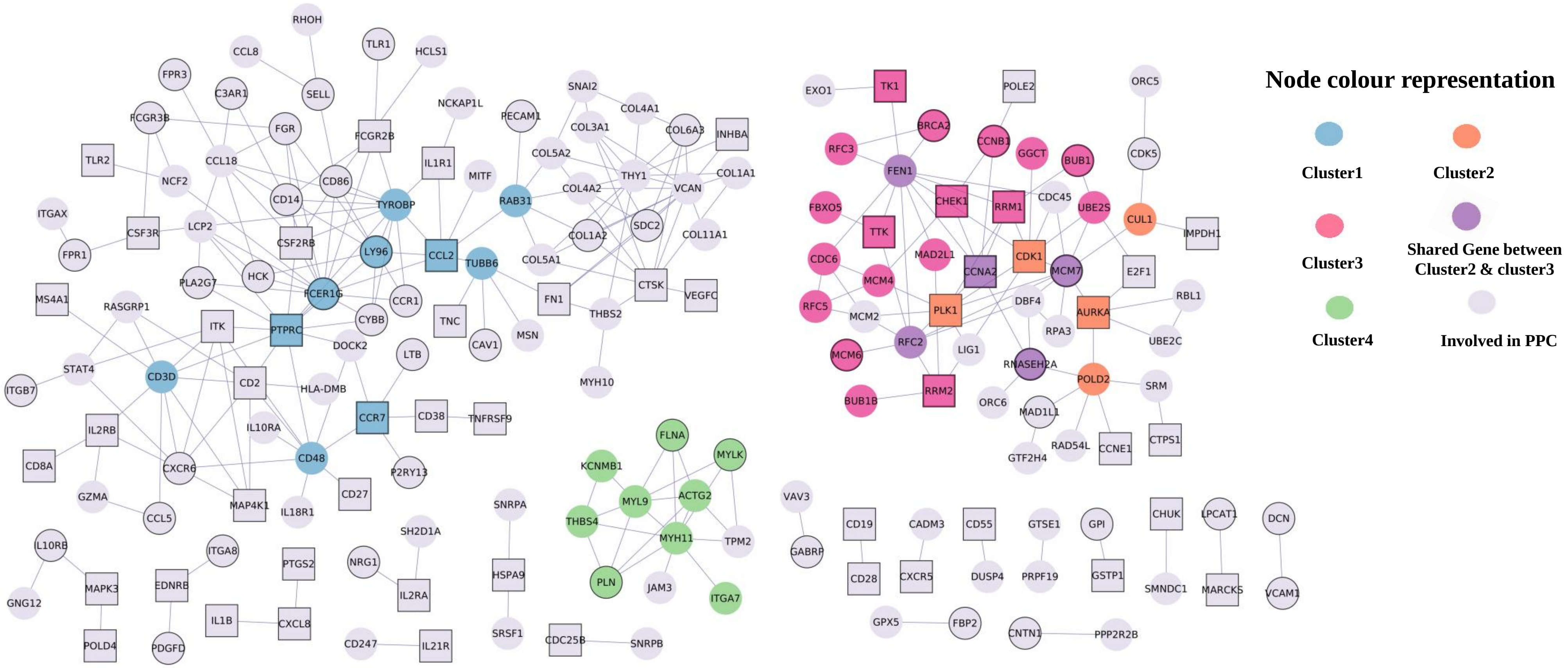


**Figure S18.** The gene co-expression network involved in pathway cross-talk in CRC state. The node with black boundaries are representing the gene used as drug targets in TTD, whereas rectangular nodes in black boundaries are the targets for at least one type of cancer.
